# Genome-Wide Identification, Characterization and Phylogenetic Analysis of the Rice LRR-Kinases

**DOI:** 10.1371/journal.pone.0016079

**Published:** 2011-03-08

**Authors:** Xinli Sun, Guo-Liang Wang

**Affiliations:** 1 College of Life Science, Hebei Normal University, Shijiazhuang, China; 2 Department of Plant Pathology, Ohio State University, Columbus, Ohio, United States of America; Kyushu Institute of Technology, Japan

## Abstract

LRR-kinases constitute the largest subfamily of receptor-like kinases in plants and regulate a wide variety of processes related to development and defense. Through a reiterative process of sequence analysis and re-annotation, we identified 309 LRR-kinase genes in the rice genome (Nipponbare). Among them, 127 genes in the Rice Annotation Project Database and 85 in Refseq of NCBI were amended (in addition, 62 LRR-kinase genes were not annotated in Refseq). The complete set of LRR-kinases was characterized. These LRR-kinases were classified into five groups according to phylogenetic analysis, and the genes in groups 1, 2, 3 and 4 usually have fewer introns than those in group 5. The introns in the LRR domain, which are highly conserved in regards to their positions and configurations, split the first Leu or other amino residues at this position of the ‘xxLxLxx’ motif with phase 2 and usually separate one or more LRR repeats exactly. Tandemly repeated LRR motifs have evolved from exon duplication, mutation and exon shuffling. The extensive distribution and diversity of the LRR-kinase genes have been mainly generated by tandem duplication and mutation after whole genome duplication. Positive selection has made a limited contribution to the sequence diversity after duplication, but positively selected sites located in the LRR domain are thought to involve in the protein-protein interaction.

## Introduction

Plant receptor-like kinases (RLKs) are transmembrane proteins with putative amino-terminal extracellular domains and carboxyl-terminal intracellular protein kinase domains [1]. The RLK family is a superfamily in plants with at least 610 members in *Arabidopsis* and about 1132 members in rice [2]. The phylogenetic-based analysis of the *Arabidopsis* RLKs provided 44 subfamilies [1]. On the basis of the extracellular domains, at least 21 different domains were found [3], and the leucine-rich repeat (LRR) domain was the largest subfamily, with 239 members in *Arabidopsis* [1].

The LRR is a widespread structural motif of 20–30 amino acids with conserved leucines, which build the domain by tandem repeat. The LRR domain forms curved solenoid structures that are particularly suitable for protein-protein interactions [4]. Based on the conserved sequence, the LRR motifs are classified into seven subfamilies, of which just one is plant-specific [4]. The nonconserved residues contribute to the specific interaction with other proteins [5]. The LRR domain in the majority of known LRR-kinases (with extracellular LRR domains and intracellular protein kinase; LK) do not possess any introns, but those in ERECTA, SERK and SYMRK/NORK are interrupted by introns at the first Leu of the ‘xxLxLxx’ motif [6], [7], [8]. The protein kinase (PK) domain consists of approximately 250–300 amino acid residues and is divided into 12 conserved subdomains (I–XII). It folds into a similar 3-dimensional catalytic core structure with a 2-lobed structure. The N-terminal lobe is smaller and includes subdomains I–IV, and the C terminal lobe is larger and includes subdomain VIA-XI [9].

PK imparts phosphotransfer according to a common mechanism. The smaller lobe is primarily involved in anchoring and orienting the nucleotide and the larger lobe is largely responsible for binding the peptide substrate and initiating phosphotransfer [9]. Many PKs can be strongly activated by the phosphorylation of the activation loop, and dephosphorylation can block the substrate access to the active site. Kinases that are regulated through this mechanism are commonly referred to as RD kinases and contain an Arg(R) in the subdomain VI preceding the catalytic loop. Conversely, a smaller number of kinases can be grouped into a class referred to as non-RD kinases that lack the conserved R in subdomain VI [10], [11]. The signal of pathogen recognition mediated by RLKs is usually through a non-RD kinase [12].

According to their biological functions, plant RLK family members can be classified into two broad categories [1]: the first category controls plant growth and development, while the second category is involved in plant–microbe interactions and defense responses [1]. As the biggest subfamily of RLKs, LKs can be also grouped into two categories. The first category includes CLAVATA1 [13], BRI1 [14], HAESA [15], etc., which play important roles in development and hormone perception; the second category comprises XA21, XA26, FLS2, EFR, etc. XA21 and XA26 confer resistance to *Xanthomonas oryzae* pv. *Oryzae* [16], [17], and FLS2 and EFR perceive the flagellin and EF-Tu of bacteria, respectively [18], [19]. It is noteworthy that some LKs have dual functions due to the cross-talk between disease and developmental pathways or due to the recognition of multiple ligands by a signal receptor [20]. For instance, BAK1 shows functions in plant developmental regulation, but recent data indicate that it also has a role in the initiation of innate immunity by positively regulating pathogen-associated molecular pattern signaling as a co-receptor [21], [22]. Similarly, ERECTA is involved in ovule development and resistance to bacterial wilt [8], [23].

Although the RLK families in *Arabidopsis* and rice were previously analyzed [1], [2], they were mainly analyzed by automatized algorithms of annotations. The results provided wrong annotations that should be revisited for an accurate description of the characteristics of the RLK genes or proteins in *Arabidopsis* and rice. In this study, we propose to identify the complete set of LK genes in the Nipponbare rice genome and re-annotated each of them using non automatized methods. A total of 309 LK genes identified in rice are supervised as well as their gene and protein structures are analyzed. The structure of some known LKs was analyzed and summarized previously [3], [5], but the characteristics of the whole set of LKs have not been reported yet.

## Results

### Manual re-annotation of the predicted LK genes in the rice genome

All of the LK genes obtained from the NCBI database and Rice Annotation Project Database (RAP-DB) were determined by the presence of LRR and PK domains with confidence (default *E* value). A total of 309 non-redundant LK genes, including pseudogenes, were revealed. The gene coding and protein sequences are shown in Table S1.

The initial sequence comparisons indicated that many LK sequences had been partially misannotated during the automated annotation process. Therefore, a complete, manual re-annotation and analysis of the LK gene family was undertaken to rectify the wrong or inaccurate predictions. The LK genes from the Reference Sequence (RefSeq) database (http://www.ncbi.nih.gov/RefSeq/) and RAP-DB were also obtained for the analysis, and the accession numbers of the loci in RAP-DB were used to name each gene in the study.

When our annotation results were compared with those in Refseq and RAP-DB (Table 1), a total of 129 and 147 errors (including no annotation genes) were found in the two databases, respectively. In RefSeq, 62 LK genes were not found; 41 LK genes were truncated or had missed exons; and 64 gene annotations were wrong (Table 1 and S2). In contrast, almost all the LK genes in RAP-DB were discovered and predicted with the exception of two genes on chromosome 11, which were named Os11g0173450 and Os11g0173550 based on the gene nomenclature [24]. Compared with our results, 59 LK gene-coding regions predicted in RAP-DB contained introns; 39 genes were truncated or had missed exons; and 31 genes had one or more other errors in their annotations (Table 1 and S2).

**Table 1:** Numbers of rice LK genes with annotation errors.

Annotation errors	In RAP-DB	In Refseq of NCBI
Including introns	59	13
Missed exons inside the gene	10	11
Missed exons inside the gene with other errors	8	4
gene fusion	1	
Incorrect intron/exon splice boundaries or numbers of exons	7	5
Truncated gene or wrong terminal exon or premature stop codon	29	41
Truncated gene with other errors	4	3
Wrong start	2	5
Wrong start and other errors	7	4
No annotation	2	62
Total	129	147

In order to comparatively analyze the RLK family in *Arabidopsis* and Rice, Shiu et al. provided an annotation for all the RLK genes in 93-11 [2]. Among them, 384 LK genes in such study were used for comparison with our annotations. Because the orthologs between *Indica* and *Japonica* rice exhibit more similarity than do paralogs [25], most of the orthologs between 93-11 and Nipponbare should have similar gene structure and sequence. The comparison showed that this group contained 114 truncated or exon-missing genes, 17 genes with introns in the predicted coding sequence and 24 gene annotations with more than one error. We also found 11 protein kinase genes, 13 LRR genes and some genes encoding proteins with unknown domains in this subfamily.

### LK gene structure and Intron/Exon configurations

**Figure 1:**
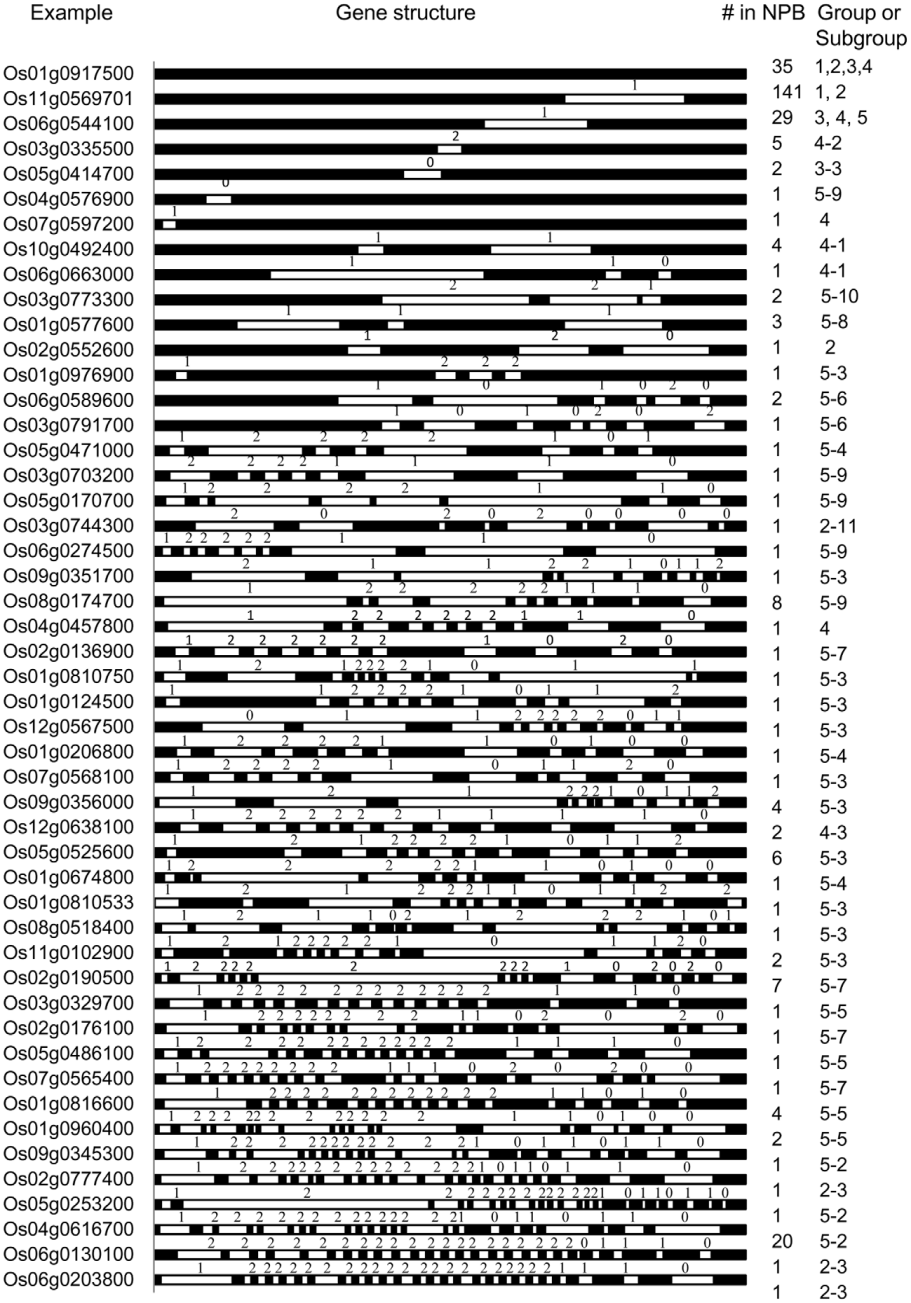
Gene structure and Introns/Exons of LK-encoding genes in rice. Introns and exons were drawn to scale with the full encoding regions of their respective gene. Filled boxes indicate the exon, and white boxes indicate the intron. Numbers under “# in NPB” indicate the number of LKs with this structure in the Nipponbare genomic sequence. 0 = Intron phrase 0; 1 = Intron phrase 1 and 2 = Intron Phase 2. *The first exon of Os01g0810533 is too small to be shown.

We analyzed the gene structure, intron positions and phases of 309 LK genes in Nipponbare. Forty-nine kinds of gene structures were found, but more than half of the LKs had simple gene structures with only one or two exons (Fig. 1). Seventy-five genes had more than 10 exons, and the most complex genes had 27 exons (Fig. 1). Os11g0569701 was the most common gene structure (141 genes), consisting of 2 exons and an intron, which inserted the GAA or GAG codon between G-A in the PK domain. One hundred forty-one genes had this kind of gene structure. Os01g0917500 was the second most common structure (35 genes), presenting only 1 exon (Fig. 1).

**Figure 2:**
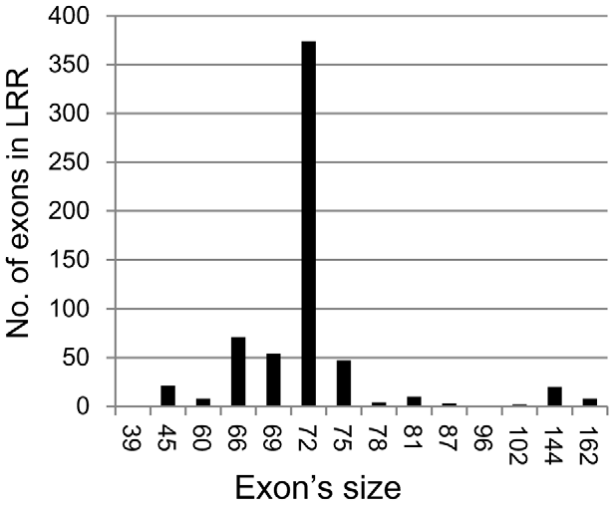
The distribution of the different sizes of exons in the LRR region.

The genes with complex structures usually consisted of some short exons in the LRR region, and most of them were interrupted by a phase-2 intron containing 72 nucleotides that only encoded 1 LRR repeat. Other exons in the LRR region encoded one or two LRR repeats (162, 144, 81, 78, 75, 69, 66, 45 and 39 nucleotides), except for the smallest exons at the end of the domain (45 and 39 nucleotides) (Fig. 2). These introns usually split the codon at the first Leu position in the known motif of ‘xxLxLxx’. The gene structures of all LKs in the LRR region were conserved. However, the position of the intron in the PK domain was poorly conserved. There were at least 26 positions that were interrupted by an intron in the PK domain; only two positions in the motif IV (P5) and VIII (P7) (date not shown) were split by introns more frequently. Conserved regions in the other regions of LKs were not found.

### Phylogenetic analysis of the rice LK proteins

**Figure 3:**
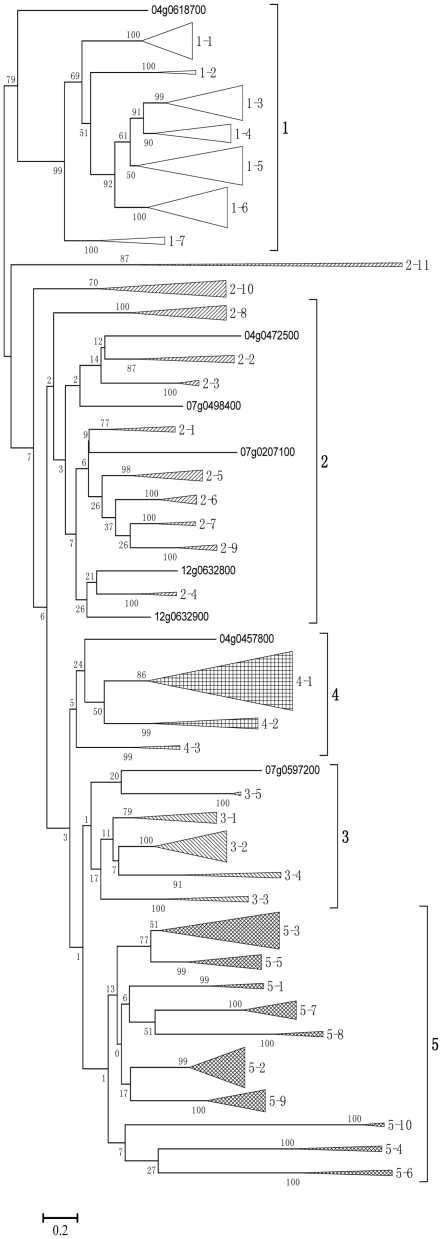
Phylogenetic trees of protein kinases of rice LKs. A ML tree with the Jones-Taylor-Thornton amino acid change model was generated with the protein kinase domain aligned with DIALIGN2. The distance scale is under the figure, and branch lengths are proportional to genetic distance. The LKs were classified into 5 groups, and group and subgroup names are shown on the right. The accession numbers of the LKs in the same subgroup that cannot be shown are replaced by the name of the subgroup.

The LK family in rice consists of many subfamilies [2]. To further elucidate the relationship among the LK proteins, the PK domains of LKs were used to generate a multiple alignment and a neighbor-joining (NJ) phylogenetic tree with Cluster X and MEGA4, respectively. From the values obtained in the bootstrap analysis, it was apparent that most of the deep nodes of the tree have low statistical significance. In order to obtain a statistically supported phylogenetic tree, two other alignment programs, MultAlin and DIALIGN2, were used to generate NJ phylogenetic trees, but the results were similar to those obtained with ClustalX and also showed low statistical values at the deep nodes (data not shown). The alternative methods of phylogenetic tree construction, minimum evolution and maximum likelihood, were tested, but the support for the minimum evolution tree was not sufficient (data not shown). However, we found the topology of the maximum likelihood (ML) trees based on DIALIGN alignment was a better fit with the gene structures, and more LK genes with similar structures were grouped together. The analysis classified the LK proteins into 5 groups. Each group was further classified into many subgroups (Fig. 3, S1). Figure S1B also shows an NJ phylogenetic tree generated with MultAlin to compare with the ML tree.

It is difficult to infer evolutionary relationships between the different groups and subgroups of LKs because the internal nodes were not well-supported, but group 1 is an exception. Group 1 was the largest group and contained 99 LK proteins, including the orthologs of *Xa21* and *Xa26* families, and all of them (with the exception of Os06g0667000, which had only 1 exon) had 2 exons (Fig. 1). Group 1 was stable and remained intact in all the trees studied, and all the nodes—even deep nodes—usually showed high bootstrap values. This finding implies that evolutionary relationships between the different subgroups in group 1 were clear (Fig. S1). Group 2 contained 51 LKs, and most of these had the same gene structure as those in group 1 with the exception of Os08g0148300, which split the GTA codon between G-T. In addition, there were 3 LKs with 1 exon; one LK (a fusion protein) with four exons; one LK with 10 exons; and 3 LKs in subgroup 2–3 with complex gene structures with 23, 26 and 27 exons, respectively (Fig. 1). Group 3 had 32 members, and most of them had only 1 exon. Four members in this group had 2 exons, but the intron split site was different from the members in group 1 (Fig. 1). Group 4 included 41 members. Genes with 1, 2, 3, 12 and 14 exons were found in this group, and the gene structures with two exons were novel compared with those of other groups (Fig. 1). Group 5 consisted of 86 members, and most of these had novel gene structures with more than 10 exons (Fig. 1). There were 35 types of gene structures in group 5 compared with 7 in group 4, 7 in group 2, 4 in group 3 and 2 in group 1 (Fig. 3).

To predict the function of LK genes, we analyzed the orthologs or the most similar homologs of some known LK genes in rice (Table S3). We found that most orthologs of resistance genes were in group 1, which included *Xa21*, *Xa26*, *FLS2* and *EFR*. Some orthologs that participate in plant development and growth were in groups 2, 3 and 4 with the exception of PEPR1, which is the receptor for AtPep1 and can induce innate immunity in response to pathogen attacks [26]. Two categories of LKs were found in group 5 (Table S3). These findings suggest that LKs in different groups show different functions: group 1 is usually involved in plant-microbe interaction and defense response; group 3 and 4 relate to plant growth and development; and group 2 and 5 are involved in both functions.

### LK protein structure

LKs usually have an LRR domain, a trans-membrane (TM) domain and a PK domain. The 309 re-annotated LK genes were translated and subjected to protein domain and motif analyses. The signal peptide, LRR, TM and PK domains were analyzed individually. These analyses are described below and are arranged from the N-terminus to C -terminus.

### Signal peptide

Using SignalP to search for the possible signal peptides in the LKs, we found that there were 84.8%, 96.5%, 90%, 95.4% and 90.8% LKs with a signal peptide in group 1, 2, 3, 4 and 5, respectively. The typical structure of signal peptides usually has a positively charged n-region, followed by a hydrophobic h-region and a neutral but polar c-region, and the cleavage sites should follow the (−3, −1) rule, which states the residues at positions −3 and −1 (relative to the cleavage site) must be small and neutral for cleavage to occur correctly [27]. Most of the signal peptides in LKs had a n-region that was usually positively charged by Arg and Lys, but about 27% (75 signal peptides) were not positively charged and 3.6% (10 signal peptides) were negatively charged. The amino acid composition in signal peptides and at the −3 and −1 positions were imbalanced and included more Leu, Ala and Val. Ninety-four percent of signal peptides had a middle helix and a two-end or one-end (N-end) coil structure, and 5.8% lacked or had a very short middle helix structure. These results suggest that the middle helix is conserved and important for LK proteins to pass through the membrane.

We used MEME to look for motifs in the signal peptide and the sequence before the LRR (including the leucine-rich repeat N-terminal domain-LRRNT). Four motifs (S1, S2, S4 and S13) existed in most LKs, and group 1, 2, 3 and 4 usually only had these 4 motifs (Fig. S3). Motif S13 appeared in the most signal peptides, but most sites in this motif were weakly conserved. Motif S2 was the neighbor of the signal peptide, and the most conserved amino acid was Leu in this motif (Table 2 and Fig. S2, S3A). The next most common motif was Motif S4, in which Trp and Leu were the most conserved. Two Cys in motif S1 were thought to be involved in the formation of LK dimers [5]. Our results show that the last Cys, Trp and Gly in this motif were more conserved than the first Cys (Table 2 and Fig. S2). Motif S1 and S4 were replaced by S7 and S11 in subgroup 5-2, respectively (Fig. S3A). Like S1, S7 also contained two conserved Cys separated by 6 other amino acids (Fig. S2). Subgroup 5-3 had more motifs than the other subgroups, and most of these motifs were specific to subgroup 5-3 (Fig. S3). The distribution of motif patterns in each group were shown in figure S3B, and the specific motif patterns usually occurred in group 5.

**Table 2:** Major motifs in signal peptide and transmembrane region within rice LKs.

Domain	Motifs	Sequence
Partial LRRNT	S1	Cx**L**x**GV**x**C** *
Partial LRRNT	S2	D/exxA**L**LxFKxxLxxp
Partial LRRNT	S4	xx**L**xS**W**xxxxx
Signal peptide	S13	lLL**L**LLlaxxxxxxx
	T1	txxsfx**GN**xx**LC**Gxx
	T2	xxxv/s/I S/tYxd/e L/ixx**AT**n/d n/g
	T7	gxxrx**F** s/t xx**EL**xxAT
TM domain	T9	x v/i xx v/i xxxxxxlxlx
	T16	x**L**h**L**px**C**xxxs

*If the bits value (see fig. S2) of the amino acid at this position is smaller than 0.5, it is represented with x; 1> bits ≧0.5, with lowercase; 2> bits ≧1, with capital letter; 3> bits ≧2, with bold capital; bits >3, with underlined capital letter in bold.

### LRR

About 26 LRR-related motifs were found in LKs (Table 3 and fig. S2). The rice LRR motifs were similar to the typical plant motif [4], but no motif was identical to this one (Table 3). The most conserved amino acid residues in rice LRR motifs were Gln at position 9, Gly at position 13 and Pro at position 16, but the Gly at position 13 was less conserved in the typical plant LRR motif [4] (Table 3). Based on the structure of all rice motifs, we concluded that the basic LRR motif should be LxxLxLxxNx L/f xGx I/l Pxx l/i Gx L/c xx.

**Table 3:** Major motifs in predicted LRR domain.

Motifs	18	19	20	21	22	23	24	1	2	3	4	5	6	7	8	9	10	11	12	13	14	15	16	17	18	19	20	21	22	23	24	1*
	x	L	G	X	L	x	x	L	x	X	L	x	L	x	x	N	x	L	t/s	g	x	I	P	x	x	L	G	x	L	x	x	L
L1	x**	l/f/i	G	X	**L**	x	x	**L**	x	X	**L**	**D**	**L**	**S**	x	**N**	x	**L**	t/s	**G**	x	**I**	**P**	x								
L2	x	l/i	G	X	**L**	x	x	**L**	x	X	**L**	x	**L**	x	x	**N**	x	**L**	S	**G**	x	**I**	**P**	x								
L3					l	x	x	**L**	x	X	**L**	n/d	L	**S**	x	**N**	x	L/f	x	**G**	x	I/v	**P**	x	x	x	x	x				
L4	x	L/i	G	X	**L**	x	x	**L**	x	X	**L**	x	**L**	x	x	**N**	x	L/f	t/s	**G**	x	**I**	**P**	x								
L5	x	L/i	x	X	**C**	t	x	**L**	x	X	L	x	L	x	x	**N**	x	L/f	x	**G**	x	I/l	**P**	x								
L6									x	x	**L**	x	L	x	x	**N**	x	F/l	t/s	**G**	x	I	**P**	x	S	L/i	g	n	l	x	x	**L**
L7	x	l/i/f	x	X	**L**	x	x	**L**	x	x	**L**	x	**L**	x	x	**N**	x	L/f	s	**G**	x	I	**P**	x	x							
L8					x	x	x	**L**	x	x	**L**	x	**L**	x	x	**N**	x	L/f	S	**G**	x	I	**P**	x	x							
L9	x	L/i	G	N	L	t	x	**L**	x	x	**L**	x	L	x	x	**N**	x	L	x	**G**	x	I/l	**P**									
L10	x	L	x	X	C	x	x	**L**	x	x	L	x	L	x	x	**N**	x	L/f	x	**G**	x	l/i	**P**	x	x							
L11					L	P	x	**L**	x	x	L	x	L	x	x	**N**	x	F/l	x	**G**	x	I	**P**	x								
L12	x	l/i	x	N	l	s	x	**L**	x	x	L	x	L	x	x	**N**	x	**L**	x	**G**	x	I	**P**	x								
L13									x	x	**L**	x	**L**	x	x	**N**	x	L/f	x	**G**	x	I	**P**	x	x	I/l	G	x	L	x	x	**L**
L14	x	L	x	X	L	x	x	**L**	x	x	L	x	L	x	x	**N**	x	L	x	**G**	x	l	**P**	x								
L15				X	x	x	x	**L**	x	x	L	x	L	x	x	**N**	x	F/L	x	**G**	x	i/l	**P**									
L16	x	L	x	X	l	x	x	**L**	x	x	**L**	x	**L**	x	x	**N**	x	**L**	x	**G**	x	l	**P**	x								
L17				X	x	x	x	L	x	x	L	x	**L**	s	x	**N**	x	**L**	x	**G**	x	I/l	**P**									
L18									x	x	L	x	L	x	x	**N**	x	L	x	**G**	x	l/i	**P**	x	x	i/l	g	x				
L19		l/i	G	X	L	x	x	**L**	x	x	L	x	L	x	x	**N**	x	L	x	x	x											
L20	x	x	g	X	L	S	s	L	x	x	L	x	l	x	x	**N**	x	L	s	**G**	x	I/l	**P**									
L21	x	L	x	X	x	x	x	**L**	x	x	L	x	L	x	x	**N**	x	L	x	**G**	x	I/l	**P**	x								
L22					x	x	x	L	x	x	L	x	L	x	x	**N**	x	**L**	x	**G**	x	I/l	**P**									
L23										x	**L**	x	L	x	x	**N**	q	**L**	x	**A**	x	x	x	x	D/g	**W**	e	**F**	l/m			
L24				x	l	x	x	L	x	x	**L**	x	L	s	x	**N**	x	L	x	**G**	x	I/l	**P**									
L25							r	**V**	t	a	L	x	**L**	x	g	x	x	**L**	x	**G**	x	L/i	**x**	P	x	l						
L26	x	L/i	x	n	x	x	x	**L**	x	x	l/i	x	L	x	x	**N**	x	F/L	x	G	x	i/l	**P**	x								

*The plant LRR motif and amino acid position in the motif come from Bella et al. (2008).

**If the bits value (see fig. S2) of the amino acid at this position is smaller than 0.5, it is represented with x; 1> bits ≧0.5, with lowercase; 2> bits ≧1, with capital letter; 3> bits ≧2, with bold capital; bits >3, with underlined capital letter in bold.

Unlike other regions in the LKs, LRR motif patterns were variable (Fig. S3A). Almost all proteins had different LRR motif patterns, except some LKs in group 5-2 that had regular motif patterns (Fig. S3A). Nevertheless, we could find some conserved regions by carefully comparing the motif patterns among different proteins, especially in the same group or sub-group. For example, the LKs in group 1 had more LRR motifs, and motif L9 and L25 were usually located at the N-terminal region and L3 was located at the C-terminus. The diversity of motif patterns in group 2 was more than other groups, and almost no conserved parts were found with the exception of motif L3 at the C-terminal region of the LRR domain. The LKs in group 3 also had the L9/25 start and L3 end patterns. Subgroup 3-2 was the largest one in group 3 and showed more regular repeat patterns than the other subgroups. The LKs in group 4 had fewer LRRs than those in groups 1, 2 and 3, and the motif patterns were less regular like group 2. However, many LKs in this group had motif L3 at the end of LRR domain. The LKs in group 5 had the least motifs of all groups (Fig. S3A).

### Transmembrane

The TM domains were predicted with TMHMM. A total of 283 LKs had at least one TM, but there were 13 LKs with more than one and 26 LKs in which any TM was not found. A single LK cannot show more than one TMs because this would place the LRR domain and protein kinase domain on the same side of the membrane. Probability values and relative positions were then used to select between predictions. Further analysis of the 26 LKs with Phobius revealed the possible TM domain in the 20 LKs, but TMHMM can also find the TMs if we lower the cutoff value. Thus, these LKs should contain atypical TMs.

Like the composition of the signal peptide, the amino acid composition of the TM domain was mainly Leu, Val, Ile and Ala . The secondary structures of the domains were predicted with HNN, and most of them showed coil and helix structures.

The motifs between the LRR and PK domains (including TM) were revealed with MEME (Table 2, Fig S2). The motif patterns were shown in Fig. S3. Motif T9 was located in the TM domain. The motifs T3, T4, T5, T6, T10, T12, T13, T14, T16 and T17 were on one side, and T2, T14, T7, T13 and T19 were on another side of the TM. They had many charged amino acid residues. As shown in figure S3B, the specific motif patterns to each group were revealed.

### Protein kinase

The PK domain in the LKs was defined by a Pfam search. About 20 motifs were found in the domain with MEME; these are named P6, P10, P3, P15, P5, P4, P2, P9, P7/16, P1, P11/12/19, P13, P8, P7/16, P14 and P19/20 from the N-terminus to the C-terminus. The motifs P6, P10, P3, P4, P2, P9, P7/16, P1 and P8 correspond to subdomains I, II, III, V, VIB, VII, VIII, IX and XI, respectively, according to their position in the PK domain and their conserved amino acid residues (Table 4) [9]. Subdomains IV, X and XII were poorly conserved, so motif P5 corresponds to subdomain IV only according to its position. It is difficult to determine the correspondence of subdomain X or XII.

**Table 4:** Major motifs of protein kinase domain in predicted rice LKs.

Subdomain*	Motifs	Sequence	Corresponding motif sequence in human PK***
IX	P1	Ks/g**DVY**/f**SF**/y**G**V/iV/l**LLE**ll**TG**K/RxPx**	Yxxxx**D**v**WS**x**G**V/ixLyE; xx**D**xWSx**G**xxx
VIB	P2	xI/vv/i**H**R/c**D**I/l/v**K**s/pS**N**I/V**LLD**	**H**xxxIi/vH**RD**L/i**K**Pe**N**i/e**L**; xxxi/v**HRDL**k/aax**N**i**L**l/v
III	P3	xs**F**xx**E**c/vex**L**/isxv/iR**H**R**NL**/iVxL/ixG/tx**C**xxxd	**E**ixiL/mxxLx**H**PNIV/ixL; **H**p**NI**vxl
V	P4	LdW/lxx**R**lxx**I**AlG/Dvv**A**xG/Ax**YLH**	xxQI/va/lx**G**/aL/mx**YL**
IV	P5	**LVYE**Y/fMpN**G**S**L**xxx**L**Hxx	l/iV/im**E**Yxxg**G**dLxxxL/I; v/iYL/iVFEymxxD**L**xxxl
I	P6	xxNlI/L**G**x**G**gf**G**x**VY**KG/axLxxG	L/I**G**x**GFG**x**V**; L/I**G**x**G**xFGx**V**
VIII	P7	xxxxxxxx**GT**/si**GY**i**APEY**g/axx	xtxx**GT**Px**Y**m**APE**Vl
XI	P8	lxxvlxl/v/iA/gL**xG**txxx**P**xx**RP**x**M**xe	axdL/fl/ixxl/m**L**xxdPxx**R**xt/s
VII	P9	dmx**A**/ph/kV/is/a/g**DFGLA**R/KLx	v/i**K**I/lx**DFG**LA/sr; KL/Ix**DFG**la
II	P10	x**VAV**/i**K**vLxxxx	V**A**i/v**K**; x**A**i**K**
	P11	**D**xm**F**xd/ggLsL/ir/hxy**V**xxAF/l**P**	
	P12	x**L**vx**W**V/axxxxxxxxx	
	P13	xev/iv/l**D**pxLxxx	
	P14	**VV**xk**L**xx**I**r/kxxyxxx	
	P15	xx**G**nD/e**FKA**	
VIII	P16	xxxxxx**GY**R**APE**vxxxxk/rS/tx	**P**L/vK/rWm/t**APE**s/a; V/iV**T**/sR/lW**YR**a/s**PE**l/v**L**L**G**x
	P17	**VV**a**ML**T/e**GD**ve/lv/a	
	P18	**VV**xQ**L**K/qEc**L**e/aL**E**xxR	
	P19	SsrxxseqsxVrxAxxqLxDi	
	P20	xssxsGSt/sxxefsxqxExxP	

*[9].

**If the bits value (see fig. S2) of the amino acid at this position is smaller than 0.5, it is represented with x; 1> bits ≧0.5, with lowercase; 2> bits ≧1, with capital letter; 3> bits ≧2, with bold capital; bits >3, with underlined capital letter in bold.

***The sequences of the human protein kinases come from Manning et al.(2002).

In order to compare rice motifs with the subdomains of eukaryotic PKs described by Hanks and Hunter [9], the conserved amino acid residues in each motif or subdomain are shown in Table 4. Human PKs were also analyzed with MEME [28] (Table 4). Most of the conserved amino acid residues indicated by Hanks and Hunter [9] were also invariant in rice LKs and human PKs. In addition, some specific conserved residues to rice PKs were found, such as Val, Val in motif P10; Phe and Leu in P3; Leu, Trp, Arg, Asp and His in P4; and Gly, Thr and Tyr in P7 (Fig. S2 and Table 4).

The LKs were divided into 5 groups based on the PK domain sequence. The results showed that motif P15 only occurred in group 1. P11 motif usually occurred in group 1 meanwhile P12 motif occurred in the others groups. However, subgroup 5-8 had a specific motif (P19). Motif P16 was presented in subgroups 4-1, 5-8 and 5-9 (Fig. S3A). Each group exhibited its specific motif patterns, and this provided further evidence for the classification (Fig. S3B). All LKs in group 1 and 93.1% in group 4 belonged to non-RD kinases. RD kinases represented 90%, 89% and 78.1% in groups 2, 3 and 5, respectively.

### Physical distribution of the LK genes in the Nipponbare genome and cluster formation

The physical distribution of the LK genes across the Nipponbare genome was investigated and is shown in Figure S4. The linked LK genes were grouped into a cluster when they were close to each other on the chromosome and in the same subgroup of the phylogenetic tree. According to this parameter, 139 LK genes were grouped into 32 clusters, and the remaining 170 genes represent single-gene loci (Table S4). The smallest clusters consisted of only 2 genes, and the largest cluster had 14 tightly linked genes on chromosome 11. The clusters were distributed unevenly over the 12 chromosomes (Table S4). More than 75% of genes on chromosome 11 and more than 50% of genes on chromosomes 2, 5 and 6 were in the cluster. In contrast, no clusters were located on chromosomes 3 and 7. The clusters were also unevenly distributed among the five groups, and group 1 contained 14 clusters, including 75.7% of the genes of the group, but group 4 had no clusters (Table S4).

A comparison of the physical positions of the genes and the phylogenetic analysis revealed both local and distant duplications of the LK genes. More similarity among each other in the same cluster suggested that these genes had been derived from tandem duplication. Highly similar clusters, such as the clusters on chromosomes 4, 5 and 8 could derive from an ancestral gene duplication and then tandem duplication separately (Fig. S1). This is more probably than a direct whole-cluster duplication because the genes into a cluster were more similar than between clusters (Fig. S1). Some singleton genes exhibited high similarity with the genes in the cluster, such as Os01g0228200, Os06g0272000, Os07g0132000, Os10g0207100 in subgroup1-1 (Fig. S1 and S4). These singleton genes could have experienced tandem duplication after their ancestral duplications or could have been derived from a member in the cluster through transpositional duplication.

### The role of selection in the diversity of genes after duplication

To reveal the selective pressures acting on the LK genes after duplication, we analyzed subgroups 1-1, 1-3, 1-4, 1-5, 1-6, 2-10, 3-2, 4-1, 5-2, 5-3 and 5-10 with the CODEML program in the PAML. These subgroups contained 195 members, including most clusters. The comparison of M0 and M3 indicated that ω was variable among sites in all of the subgroups (Table 5). Positively selected sites were found in subgroups 1-3, 1-4 and 1-5 but not in subgroups 1-6, 2-10 and 5-10 by all three pairs of models. Positively selected sites were also revealed in subgroups 1-1, 3-2, 4-1, 5-2 and 5-3 by testing M7 and M8, M8a and M8, but they were not found by M1a and M2a (Table 5). These results indicate that the M7–M8 and M8a–M8 comparisons appear to be more robust than the M1a–M2a comparisons for our data.

**Table 5:** Likelihood ratio test of positive selection in family proteins.

Sub-group	n^1^	2Δ*l* M3 vs. M0	2Δ*l* M2a vs. M1a	2Δ*l* M8 vs. M7	2Δ*l* M8 vs. M8a	M8estimates^3^	Positively selected sites (Posterior>0.90)^4^	Percent of positively selected sites in xxLxLxx motif to LRR domain
1-1	20	3587.8**^2^	0	185.4**	122.8**	p1 = 0.069, ω = 2.26	57, **69**, **230**, **279**, **335**, **339**, **340, 363**, **386**, **411**, **435**, **440, 464**, **484**, 511, 513, **516**, **537, 539**, **541**, **586**, **636**	37/38
1-3	19	5657.6**	413.4**	98.8**	53.6**	p1 = 0.067, ω = 1.68	**189**, **285**, 310, 315, 335, **340**, 414, 438, 509, **536**,	34/38
1-4	10	1481.2**	280.4**	14.8**	8.2**	p1 = 0.041, ω = 1.71	Posteriors of the sites <0.90	14/19
1-5	21	4145.8**	84.8**	59.4**	57.8**	p1 = 0.069, ω = 1.81	**327**, **346**, **373**, **447**, **471**, **496**, 498, **526**, 555, 575, **628**, 1162, 1216, 1217	24/27
1-6	22	2440.8**	0.0	1.2	0.0	p1 = 0.037, ω = 1.0	None	
2-10	9	1127.4**	0.0	1.7	0.0	p1 = 0.037, ω = 1.12	None	
3-2	17	2503.2**	0.0	6.2*	4.6*	p1 = 0.022, ω = 1.56	Posteriors of the sites <0.90	6/13
4-1	25	2634.6**	0.0	7.7*	4.8*	p1 = 0.016, ω = 2.36	**344**	none
5-2	22	2786.8**	0.0	120.0**	46**	p1 = 0.080, ω = 1.64	**15**, 28, **30**, **32, 33, 34, 36, 165**, 680, **1069**, **1074**, **1099**, **1107**, **1108, 1111**	3/4
5-3	20	2846.4**	0.0	24.5**	22.4**	p1 = 0.026, ω = 2.51	352, 467, 452, 446	none
5-10	11	1279.0**	0.0	0.0	0.0	p1 = 0.00001, ω = 1.0	None	

1 Number of sequences in the group.

2 *: significant at 5% level, **: significant at 1% level.

3 ω is *d*N:*d*S estimated under M8; *p*1 is the inferred proportion of positively selected sites.

4 Sites potentially under positive selection identified under model M8 are listed according to conserved sequence numbering respectively. Positively selected sites with posterior probability >0.99 are underlined in bold, 0.95–0.99 in bold.

The distribution of ω along different domains, and the ω with error bar and posterior value of the positive selection sites tested with Bayes Empirical Bayes (BEB) analysis under Model 8 are shown in figure S5 [29]. Most of the positively selected sites occurred in the N-terminal, LRR and C-terminal regions and rarely in the transmembrane and protein kinase regions. Some positively selected sites were detected in the region between LRR and the protein kinase domain, except for the TM domain. In groups 1 and 3, the positively selected sites were concentrated in the LRR domain, but in groups 4 and 5, only a few were in this domain (Fig. S5). Most of the positively selected sites in the LRR domains presented the xxLxLxx motif (Table 5).

The diversity of the LKs in each subgroup was detected by calculating the percentage differences of amino acid sites in deduced proteins (Fig. S6). Though differences in diversity were found in different subgroups, they were not correlated with positive selection. Some subgroups showed higher diversity without obvious positive selection, such as subgroups 1-6, 2-10 and 5-10 (Fig. S6 and Table 5). Most of the codons in genes are under purifying selection, and many, including some positively selected sites (ω close to 1), identified codons were under relaxed purifying or neutral selection (Fig. S5 and Table 5). In addition, the number of positively selected sites was far less than that of the polymorphic sites among the sequences (data not shown).

Comparing the positively selected sites among different subgroups, we found that these sites were usually located in the LRR domain in subgroups 1-1, 1-3, 1-4, 1-5 and 3-2, whose genes had a simple gene structure without or with 1 intron. However, in subgroups 4-1, 5-2 and 5-3, most of the positively selected sites were outside the LRR domain. In subgroups 2-10 and 5-10, no positively selected sites were detected. The members in subgroups 2-10, 5-2 and 5-3 had complex gene structures with more introns in the LRR regions. Therefore, the split LRR domain may limit the variation of the selection sites.

## Discussion

Automated annotations of sequenced genomes using computer programs can cause a high level of misannotation and misinterpretation. Previous study found that approximately one-third of the automated annotations contained errors in the NBS-LRR–encoding genes in *Arabidopsis* [30]. Our analysis revealed 41.4% of the annotations had errors in 307 LK genes in the RAP-DB database and 34.4% of the annotations in 247 LK genes had errors in the Refseq of NCBI (2 and 62 LK genes were not annotated in RAP-DB and Refseq, respectively). These results confirmed that manual annotation is necessary to study the gene structures and their evolutionary relationships, particularly when large gene families are considered.

We have carefully characterized the complete set of 309 LK genes in the Nipponbare genome. Based on gene structure and protein kinase domain sequence divergence, we defined 5 groups; this classification differed from Shiu et al.'s that divided the LKs into 15 subgroups or groups according to phylogenetic results [2]. Their results were mainly derived from automated annotations. We found that the LKs in groups 1 and 2 usually had simple gene structures with 1 intron that split the conserved Glu of motif P7 (VIII) in the PK domain. Most LKs in group 3 had no intron, and those in group 4 had 1 intron that was different from the LKs in group 1 and 2 and split an unconserved amino acid in motif P5 (IV) of the PK domain. LKs in group 5 usually had many introns. These results suggest that our classification could be more accurate. The specific protein motif patterns in each group or subgroup provide additional support for our classification (Fig. S3).

**Figure 4:**
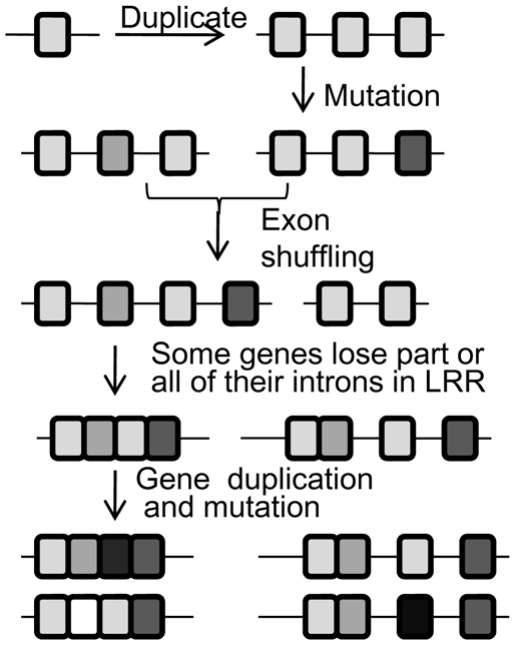
The Model for LRR domain evolution of LKs. Boxes indicate exons, and lines indicate introns.

The gene structure analysis showed that the intron position in the LRR domain was highly conserved and split the first Leu or other amino residues at this position of the motif xxLxLxx with phase 2. The intron usually separated one or more LRR repeats. Motif analysis revealed that the alignment order of the LRR repeats in different LKs was diversified (Fig. S3). The discovery of the short exon encoding one LRR repeat suggested that the LRR domain was produced from the duplication of the short exon and that exon shuffling played a major role in the diversity of the LRRs. We propose that an ancient LRR gene encoded one LRR repeat, and its duplication produced more exon-containing LRR genes; subsequent mutations and the exon shuffling produced gene diversity. Some genes may have lost the intron between the LRR repeats leading to novel genes (Fig. 4). There are at least four major mechanisms to produce duplicate genes: (1) genome duplication, (2) tandem gene duplication, (3) segmental duplication and (4) transpositional duplication [31]. The rice genome was created by a whole-genome duplication and subsequent “diploidization” (loss) of many duplicated gene copies [32], [33]. We found that at least 15 pairs of LK genes were located on the retention regions after genome duplication. The genes in the same cluster showed more similarity to each other (Fig. S1), suggesting that the genes in the cluster had been derived from tandem duplication after the whole genome duplication. About 45% of the genes in clusters seemed to indicate that tandem duplication played a major role for the formation of the gene families.

Positive selection is likely the cause of accelerated amino acid substitutions in some duplicate genes. Our results, however, showed that the number of positively selected sites was generally small, and most of sites were under stringent or relaxed purifying selection (Table 5, Fig. S5). Positive selection could not be detected in other duplicate gene groups. In addition, we could not find a correlation between diversity and positive selection (Table 5 and fig. S6), and there are many polymorphic sites in which positive selection was not detected among the duplicated genes (data not shown). Similar phenomena were also observed in *Arabidopsis's* NBS-LRR gene families [34]. These findings suggest that positive selection was not a strong factor for the sequence diversification of the duplicate genes and that most amino acid sites were under neutral or near neutral selection. Though only a small number of amino acids were affected by positive selection, these amino acids were usually located on the xxLxLxx motif of the LRR in LKs (Table 5) and NBS-LRRs [34], which is thought to directly interact with its ligand and determine the protein specificity of the binding [35]. Similar evidence was also discovered in other genes, such as the UV opsin (duplicated UVRh2) of the butterfly *Heliconius erato*, trypsin-like serine protease in mosquito, antigen CD4 of T-cells in primates and multicystatin (SlCYS8) in tomato [36], [37], [38], [39]. The change of the positively selected sites in these genes affects the proteins' activity and function.

Many models for the emergence, maintenance and evolution of gene copies have been proposed, but there is not a clear consensus among them [40]. Selection plays different roles in different models [40]. Positive selection was observed across the LRR domains among some duplicated resistance genes (Table 5) [34]. However, only stringent or relaxed purifying selection was detected in some other LK and NBS-LRR genes [34], [41]. Dangl and McDowell suggested that the NBS-LRR proteins can be classified into two types [42]. Type I, which can directly interact with the effector, are co-evolving directly and dynamically with an effector(s) and are under positive selection, whereas type II genes, which detect the host protein modified by effectors, are evolving in a more conservative mode and are under purified selection [42], [43]. This difference between proteins implies that protein function is the key factor that determines what kind of selection will be applied to the gene.

## Materials and Methods

### Sequence retrieval

In order to find all of the LK genes in the rice genome (*Oryza sativa* subsp. *japonica* cv Nipponbare), a BLAST search was used repeatedly. Firstly, protein BLASTs with a default E value cutoff against the rice Ref protein database were performed using a Hidden Markov Model (HMM) profile of LRR and the protein kinase domain as the query [44]. The common items between the LRR and protein kinase results were collected. All of these proteins were then clustered with ClustalX and divided into several groups by phylogenetic analysis with MEGA4 [45], and one or two proteins in each group or sub-group were picked as queries to perform tBLASTn searches against the rice genome sequence in order to find more LK genes. Then, all of the LK genes obtained from Nipponbare were compared with the results obtained from 93-11 [2]. Any LK gene in 93-11 for which an ortholog was not found was used as a query to search the Nipponbare genome again to reveal the undiscovered LK genes. Finally, the presence of LRR and protein kinase domains was verified by searching the Pfam HMMs with confidence (Default *E* value) [44].

### Re-annotation

All LK genes were re-annotated using the procedures detailed below. First, the gene position in the BAC was determined with Genescan (http://genes.mit.edu/GENSCAN.html) and/or BLAST to identify the genomic sequence of the candidate gene. Each LK was then used as a query to search the ESTs and the full-length cDNA database to check if the annotation results were similar to the cDNAs. When results didn't match, annotations were corrected following cDNA database information. However, we found some full-length cDNAs that contained introns that probably belong to genomic fragments (The possible reasons are that there are some DNA contaminations or some mRNAs that were not completely processed were reverse transcripted during making the cDNA library). In such cases, we used BLASTx to search for known proteins and identify the introns. We also used this method to reveal the possible frame-shift sites and missing region. Finally, for the LK genes without cDNA and ESTs matches, the predictions were manually performed as described by Meyer et al. [30].

### Alignments and phylogenetic reconstruction

For the alignment of the PK domains, complete predicted protein sequences for the LKs were trimmed according to the HMM Pfamseq of the protein kinase (PF07714) (http://pfam.janelia.org/family/PF07714#tabview=tab0). Sequences were aligned using ClustalX [46], DIALIGN 2 [47] or MultAlin [48] with default options. Phylogenetic analyses with Poisson Model, including NJ, minimum evolution, and bootstrap analyses with 1000 replicates, were performed using MEGA 4.0 [49]. Bootstrapping provided an estimate of the confidence for each branch point. The ML tree was made with FastTree program with Jones-Taylor-Thornton model of amino acid evolution [50], [51]. In order to calculate bootstrap value, Phylip's SEQBOOT (http://evolution.genetics.washington.edu/phylip/doc/seqboot.html) was used to generate resampled alignments with 500 replicates, and FastTree analyzed all of the resampled alignments.

### Protein structure analysis

The possible signal peptides in LKs were predicted with SignalP (http://www.cbs.dtu.dk/services/SignalP/). SignalP predicted the cleavage sites of signal peptides based on a combination of several artificial neural networks and hidden Markov models [52]. When both methods didn't get the same result, subjective criteria of similarity with the typical structure were used. Transmembrane domains were predicted with TMHMM (http://www.cbs.dtu.dk/services/TMHMM-2.0/) and Phobius (http://phobius.binf.ku.dk/). Programs hmmpfam and hmmsearch were used to determine the LRR domain in each LK [53]. The motifs were discovered with MEME (http://meme.sdsc.edu/meme4_4_0/cgi-bin/meme.cgi) and visualized with Logo (http://weblogo.berkeley.edu/logo.cgi). The secondary structures of the signal peptides and transmembrane domains were predicted with HNN (http://npsa-pbil.ibcp.fr/cgi-bin/npsa_automat.pl?page=npsa_nn.html).

### Positive selection analysis

The selective pressures acting on the receptor region were estimated by using the CODEML program in the PAML(4.2) package [54]. The codon alignments based on an existing protein multiple-sequence alignments for CODEML were created with the PAL2NAL [55]. The heterogeneity in ω among sites was tested by comparing M0 and M3, and the positive selections were tested with three pairs of models, i.e., M1a and M2a, M7 and M8, M8a and M8, where M1a (nearly neutral) and M2a (positive selection) were slight modifications of models M1 (neutral) and M2 (selection) in Nielsen and Yang [56]. M7 used 10 ω categories to describe ω among the sites, all constrained to be <1; M8 differed from M7 only in that it estimated ω for an extra class of sites (p10) at which ω could be >1, and M8a fixed this extra category at ω = 1. [29], [56], [57], [58]. For details, refer to the reference [59] and the user guide for PAML (http://abacus.gene.ucl.ac.uk/software/paml.html).

## Supporting Information

Figure S1 **Phylogenetic trees of protein kinases from rice LKs.**
(PPT)

Figure S2 **The motif sequence Logos in the rice LKs.**
(PPT)

Figure S3 **(A) The motif patterns in rice LKs.** (B) The distribution of the motif patterns in each group.
(XLS)

Figure S4 **Physical locations of genes encoding LKs in the rice genome.**
(PDF)

Figure S5 **The distribution of the posterior probabilities and ω values for sites along the LK proteins in different subgroups.**
(PPT)

Figure S6 **The percentage differences of amino acid sites in deduced proteins of the LKs in 11 subgroups.**
(PPT)

Table S1 **Coding DNA and protein sequences of the 309 LKs in rice.**
(XLS)

Table S2 **The accession numbers with incorrect annotations in RAP-DB and Refseq.**
(XLS)

Table S3 **The functions of some known LKs and their orthologs in rice.**
(DOC)

Table S4 **Clusters of LK genes in rice Nipponbare.**
(DOC)
