# Supplementary material for: Genome-Wide Identification, Characterization and Phylogenetic Analysis of the Rice LRR-Kinases
Source: PLoS One. 2011 Mar 8;6(3):e16079. doi: 10.1371/journal.pone.0016079 (PMC3050792; doi:10.1371/journal.pone.0016079)
Supplement: Figure S1 — Phylogenetic trees of protein kinases from rice LKs. (PPT) [file pone.0016079.s001.ppt]

## Slide 1
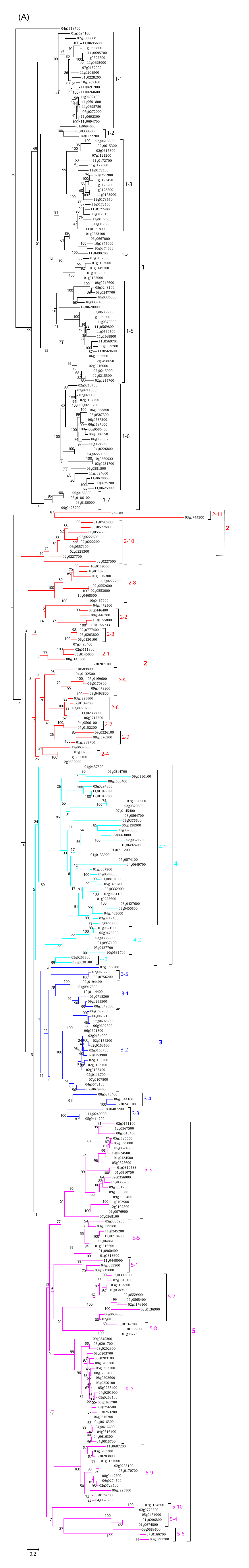

(A)
2

## Slide 2
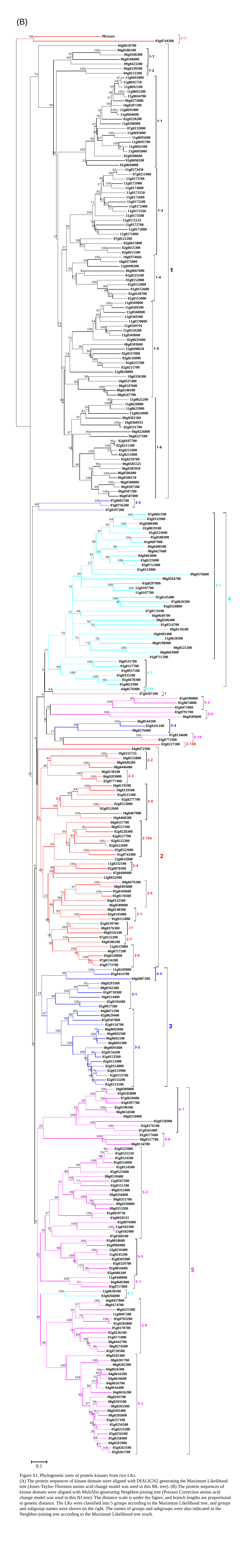

(B)
Figure S1. Phylogenetic trees of protein kinases from rice LKs.
(A) The protein sequences of kinase domain were aligned with DIALIGN2 generating the Maximum Likelihood tree (Jones-Taylor-Thornton amino acid change model was used in this ML tree). (B) The protein sequences of kinase domain were aligned with MultAlin generating Neighbor-joining tree (Poisson Correction amino acid change model was used in this NJ tree). The distance scale is under the figure, and branch lengths are proportional to genetic distance. The LKs were classified into 5 groups according to the Maximum Likelihood tree, and groups and subgroup names were shown on the right. The names of groups and subgroups were also indicated in the Neighbor-joining tree according to the Maximum Likelihood tree result.
