# Supplementary material for: Genome-Wide Identification, Characterization and Phylogenetic Analysis of the Rice LRR-Kinases
Source: PLoS One. 2011 Mar 8;6(3):e16079. doi: 10.1371/journal.pone.0016079 (PMC3050792; doi:10.1371/journal.pone.0016079)
Supplement: Figure S2 — The motif sequence Logos in the rice LKs. (PPT) [file pone.0016079.s002.ppt]

## Slide 1
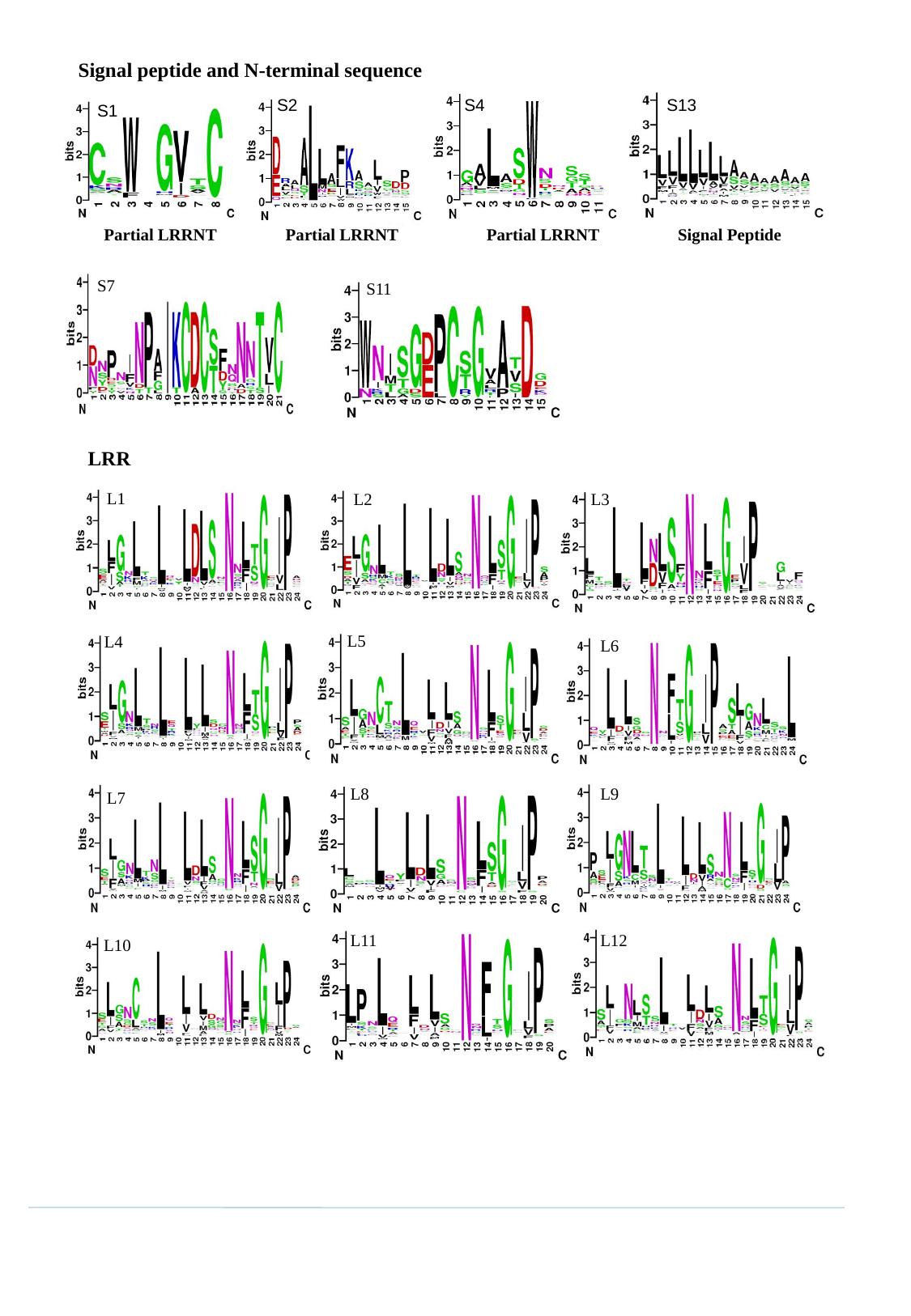

Signal peptide and N-terminal sequence
S2
S4
S13
S1
Partial LRRNT
Partial LRRNT
Partial LRRNT
Signal Peptide
S7
S11
LRR
L1
L3
L2
L5
L4
L6
L8
L9
L7
L11
L12
L10

## Slide 2
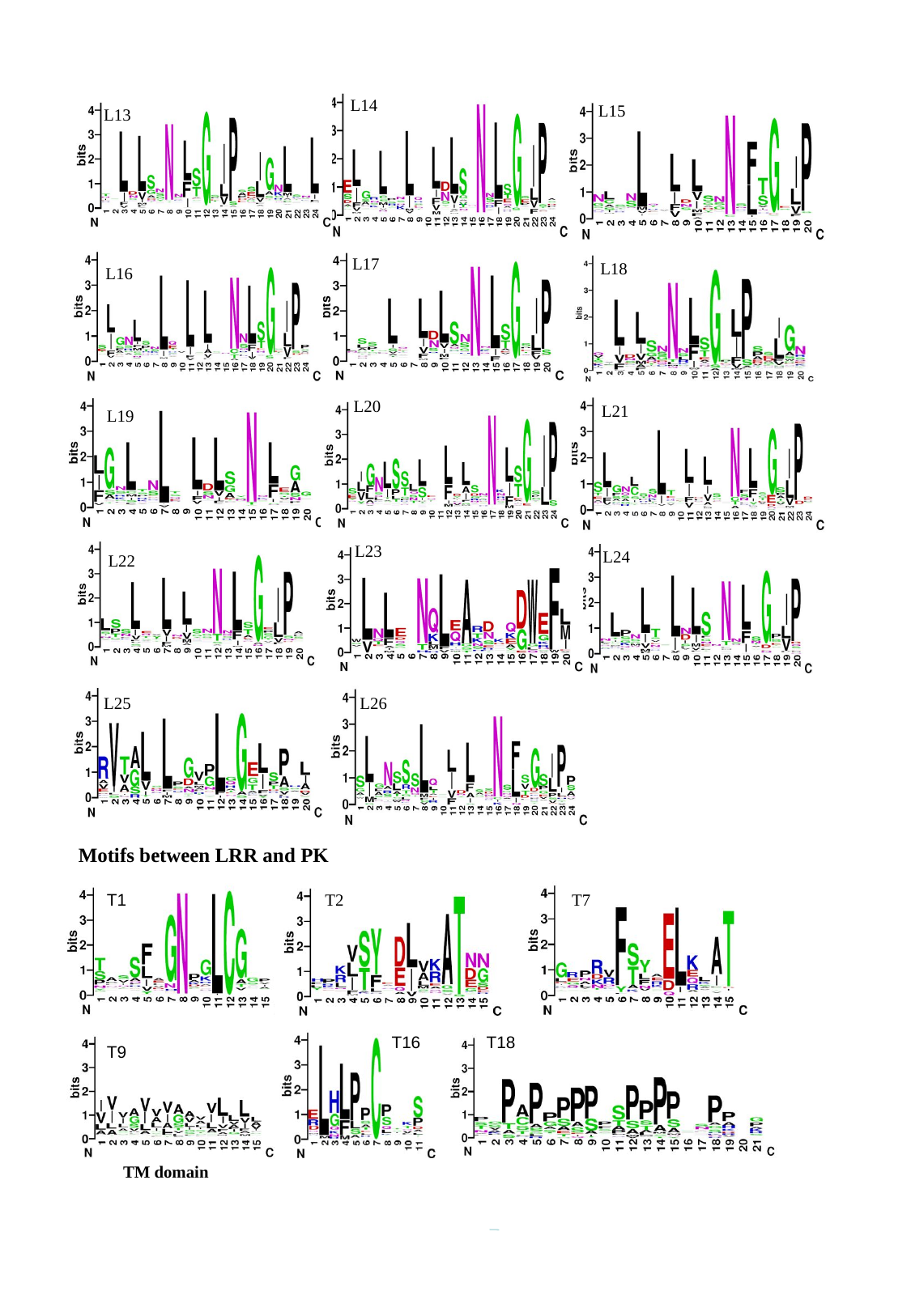

L14
L15
L13
L17
L18
L16
L20
L21
L19
L23
L24
L22
L25
L26
Motifs between LRR and PK
T1
T2
T7
T16
T18
T9
TM domain

## Slide 3
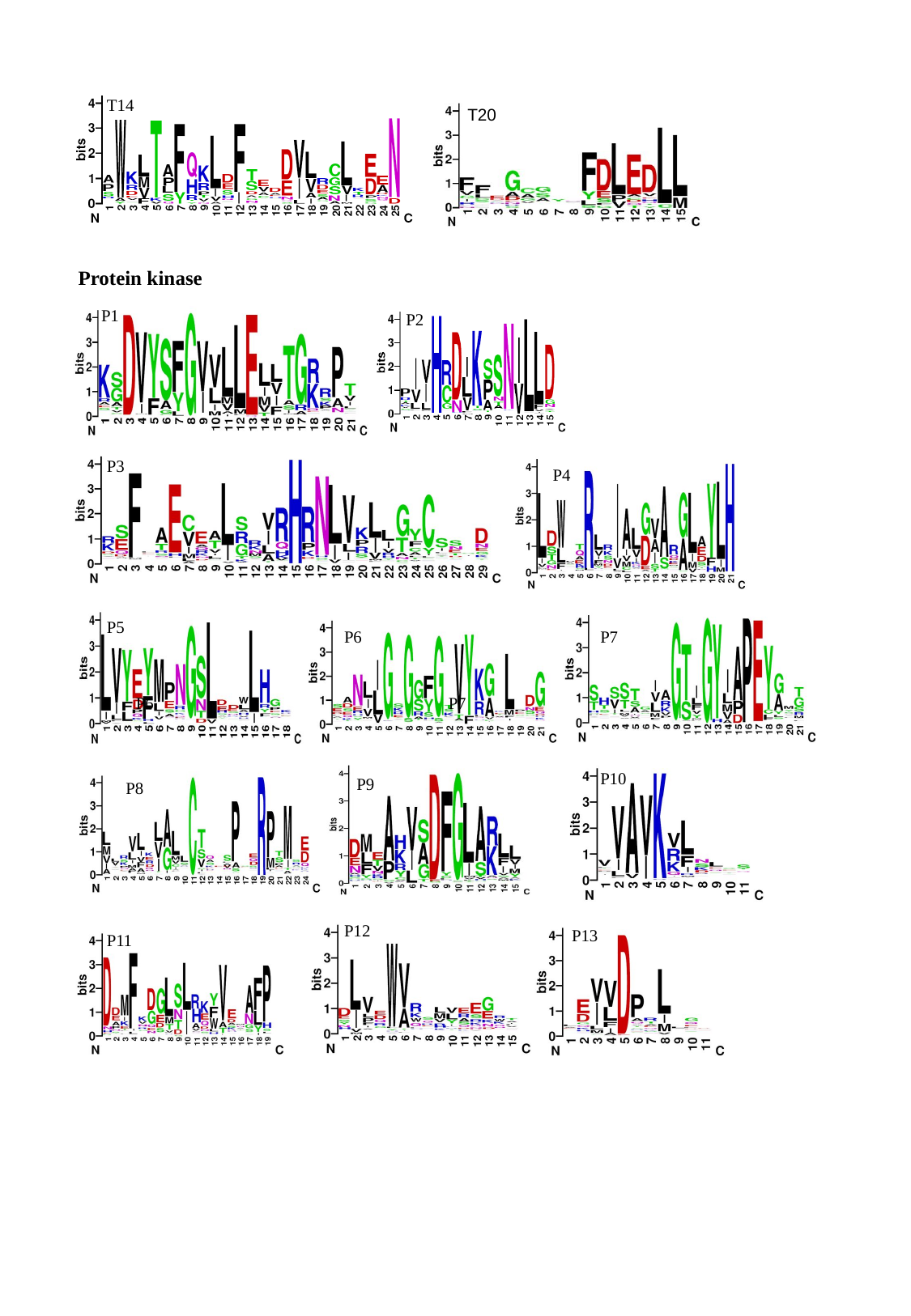

T14
T20
Protein kinase
P1
P2
P3
P4
P5
P6
P7
P7
P6
P10
P9
P8
P12
P13
P11

## Slide 4
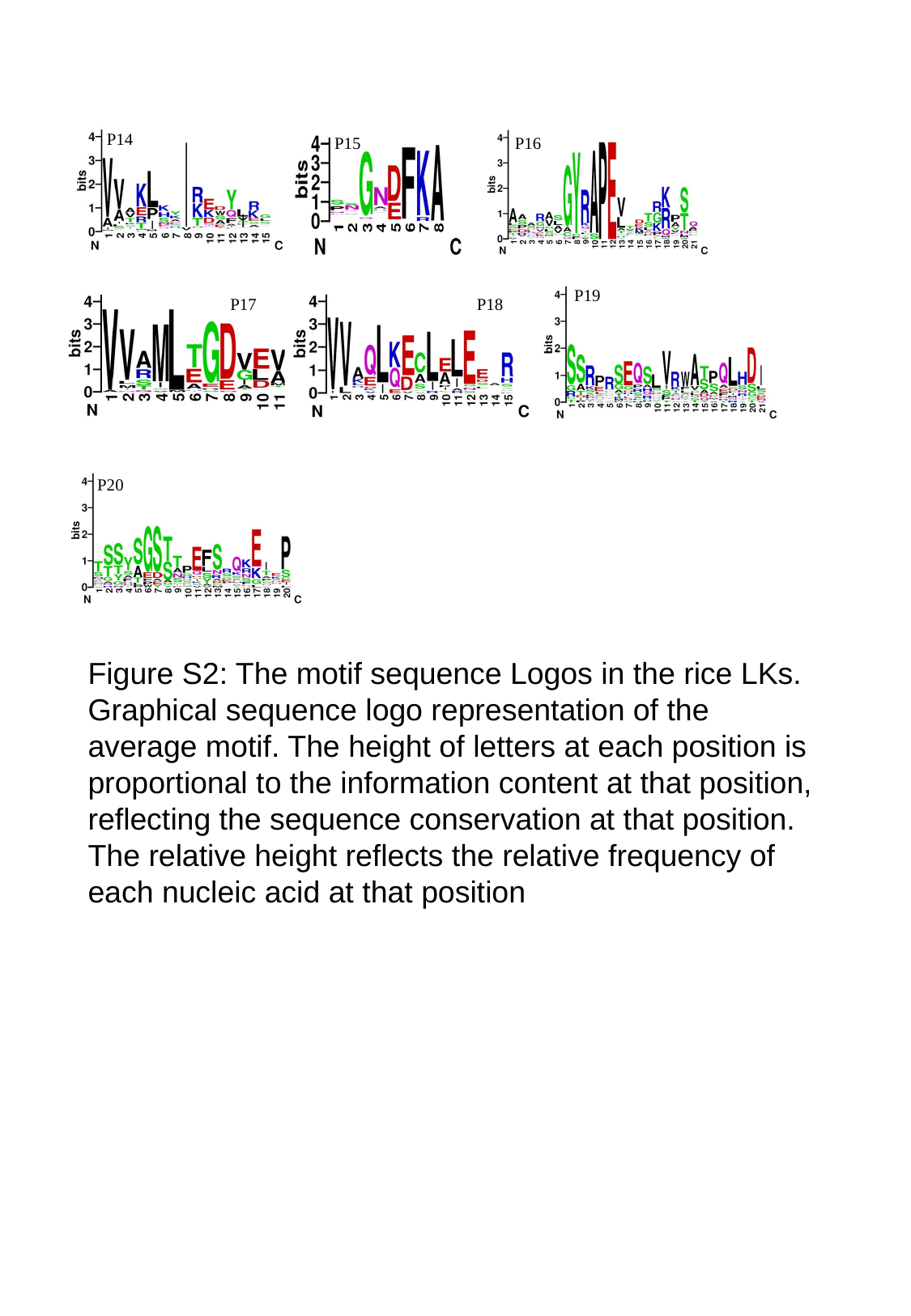

P14
P15
P16
P19
P17
P18
P20
Figure S2: The motif sequence Logos in the rice LKs.
Graphical sequence logo representation of the average motif. The height of letters at each position is proportional to the information content at that position, reflecting the sequence conservation at that position. The relative height reflects the relative frequency of each nucleic acid at that position
