# Supplementary material for: Genome-Wide Identification, Characterization and Phylogenetic Analysis of the Rice LRR-Kinases
Source: PLoS One. 2011 Mar 8;6(3):e16079. doi: 10.1371/journal.pone.0016079 (PMC3050792; doi:10.1371/journal.pone.0016079)
Supplement: Figure S4 — Physical locations of genes encoding LKs in the rice genome. (PDF) [file pone.0016079.s004.pdf]

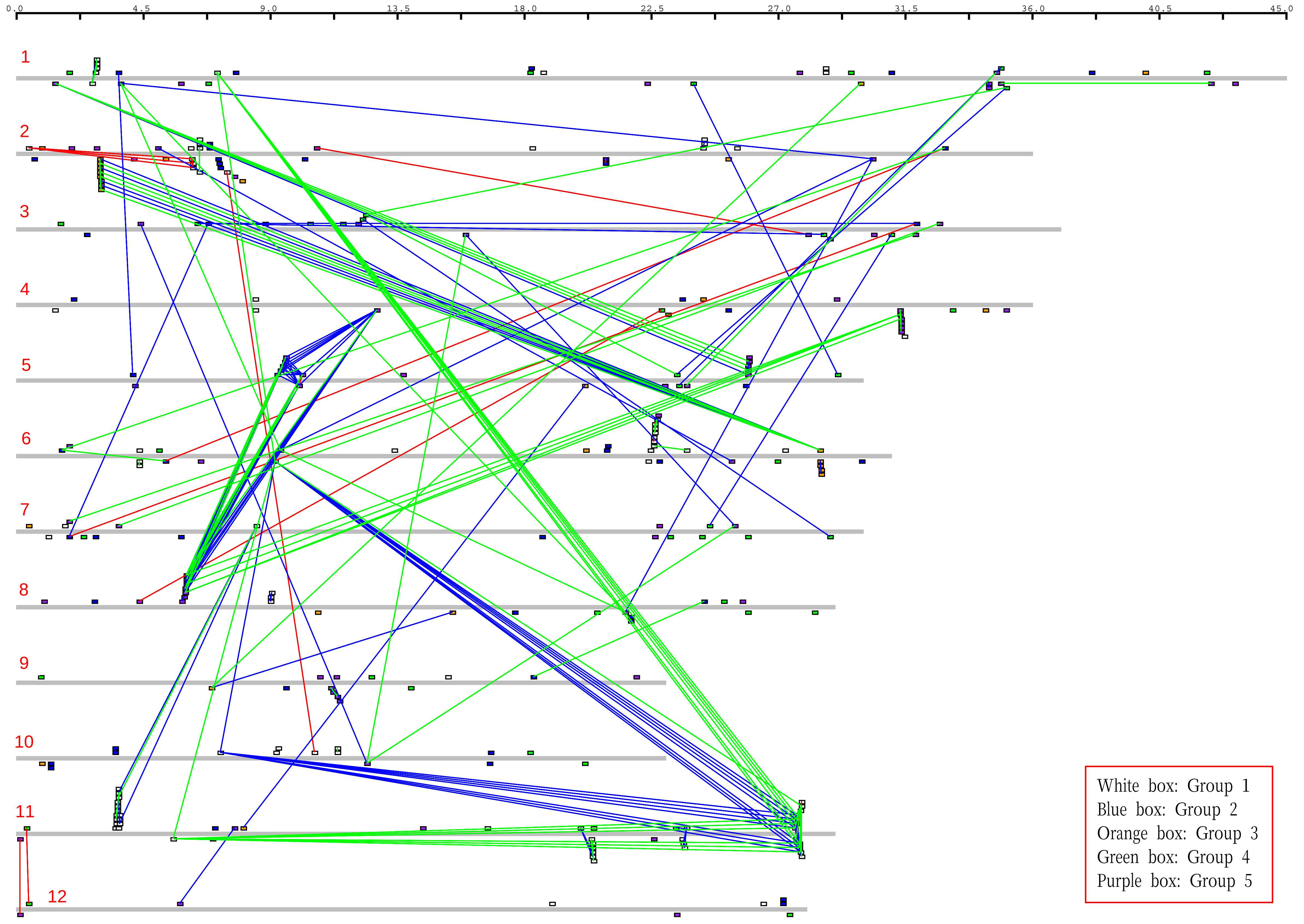

Figure S4. Physical locations of genes encoding LKs in the rice genome.

Boxes above and below each rice chromosome (gray bars) designate the approximate locations of each gene. Chromosome lengths are shown in megabase pairs on the scale at top. The lines link the LKs with the same or more than 70% protein kinase identity. Red line: ≧90%; Blue line:≧80 and <90; Green line: ≧70% and <80%.

Created with GenomePixelizer
