# Supplementary material for: Genome-Wide Identification, Characterization and Phylogenetic Analysis of the Rice LRR-Kinases
Source: PLoS One. 2011 Mar 8;6(3):e16079. doi: 10.1371/journal.pone.0016079 (PMC3050792; doi:10.1371/journal.pone.0016079)
Supplement: Figure S5 — The distribution of the posterior probabilities and ω values for sites along the LK proteins in different subgroups. (PPT) [file pone.0016079.s005.ppt]

## Slide 1
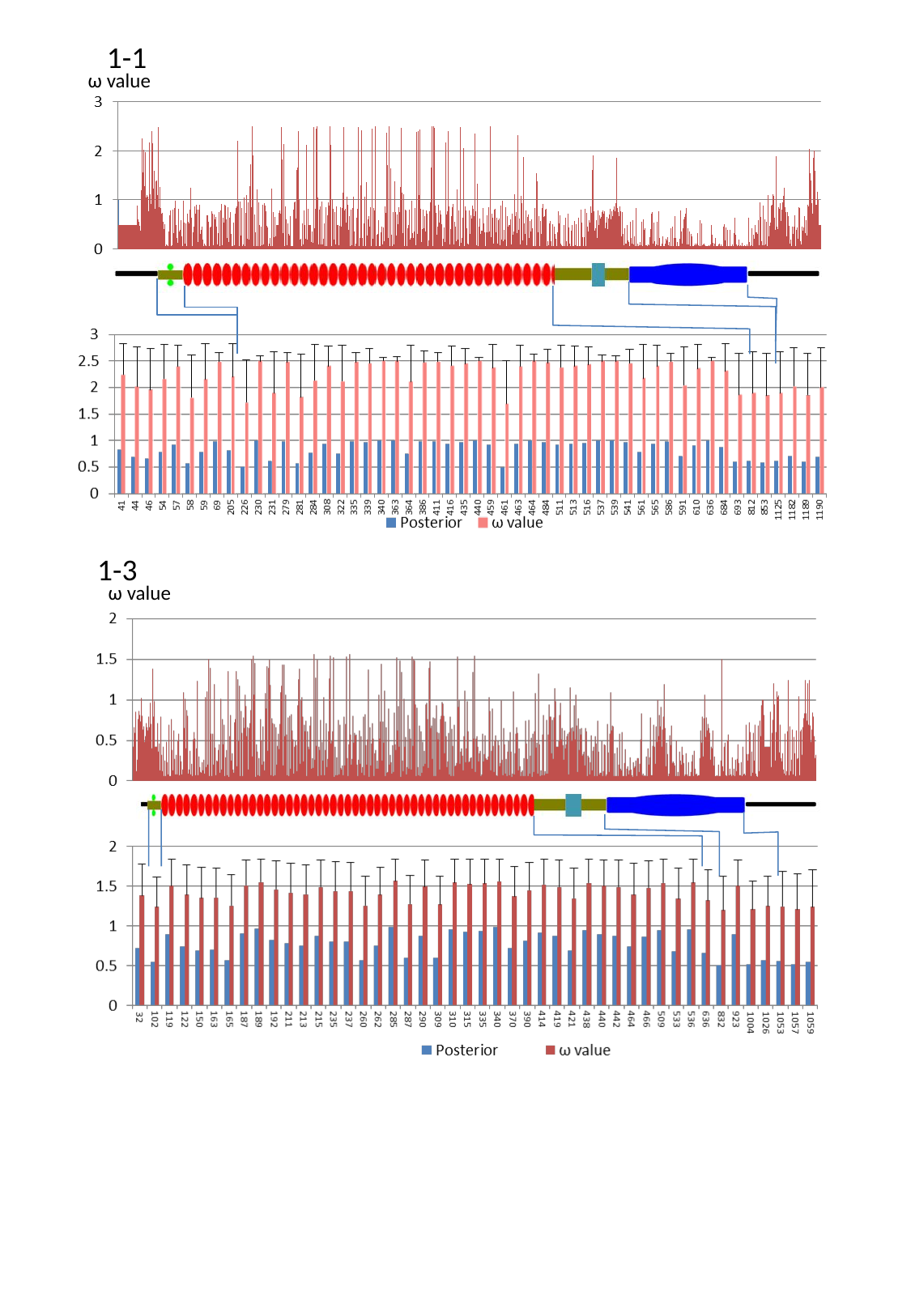

1-1
ω value
1-3
ω value

## Slide 2
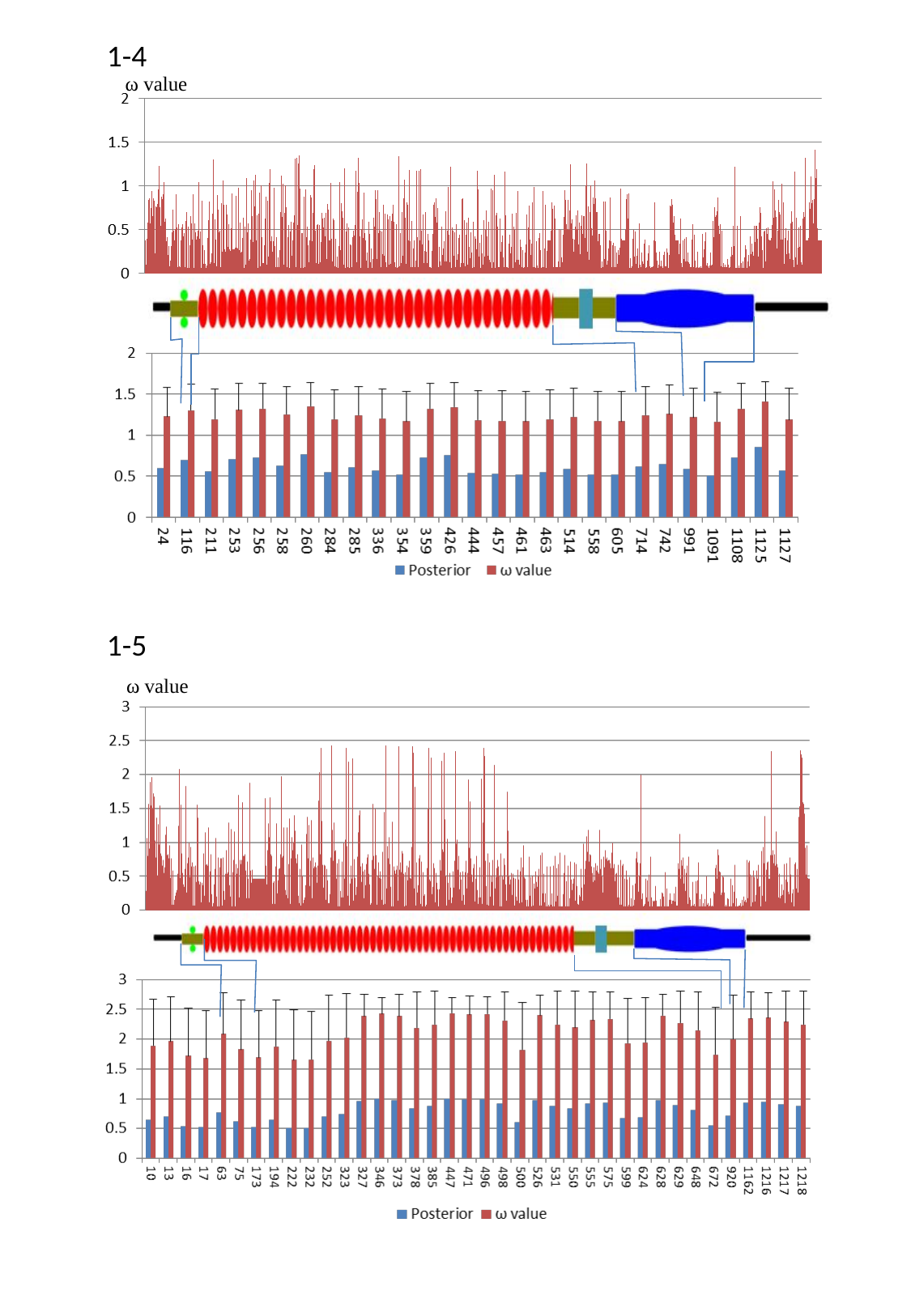

1-4
ω value
1-5
ω value

## Slide 3
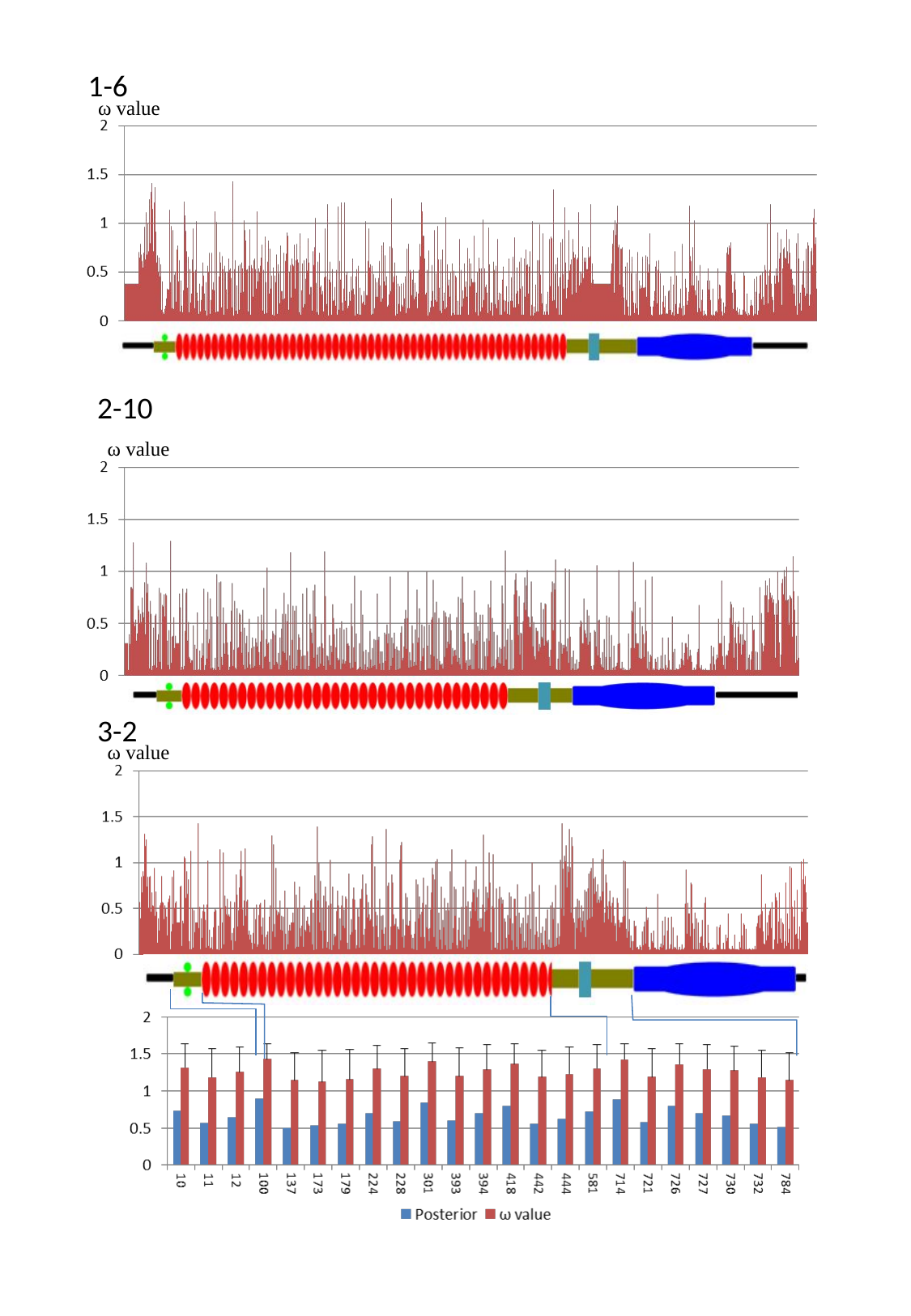

1-6
ω value
2-10
ω value
3-2
ω value

## Slide 4
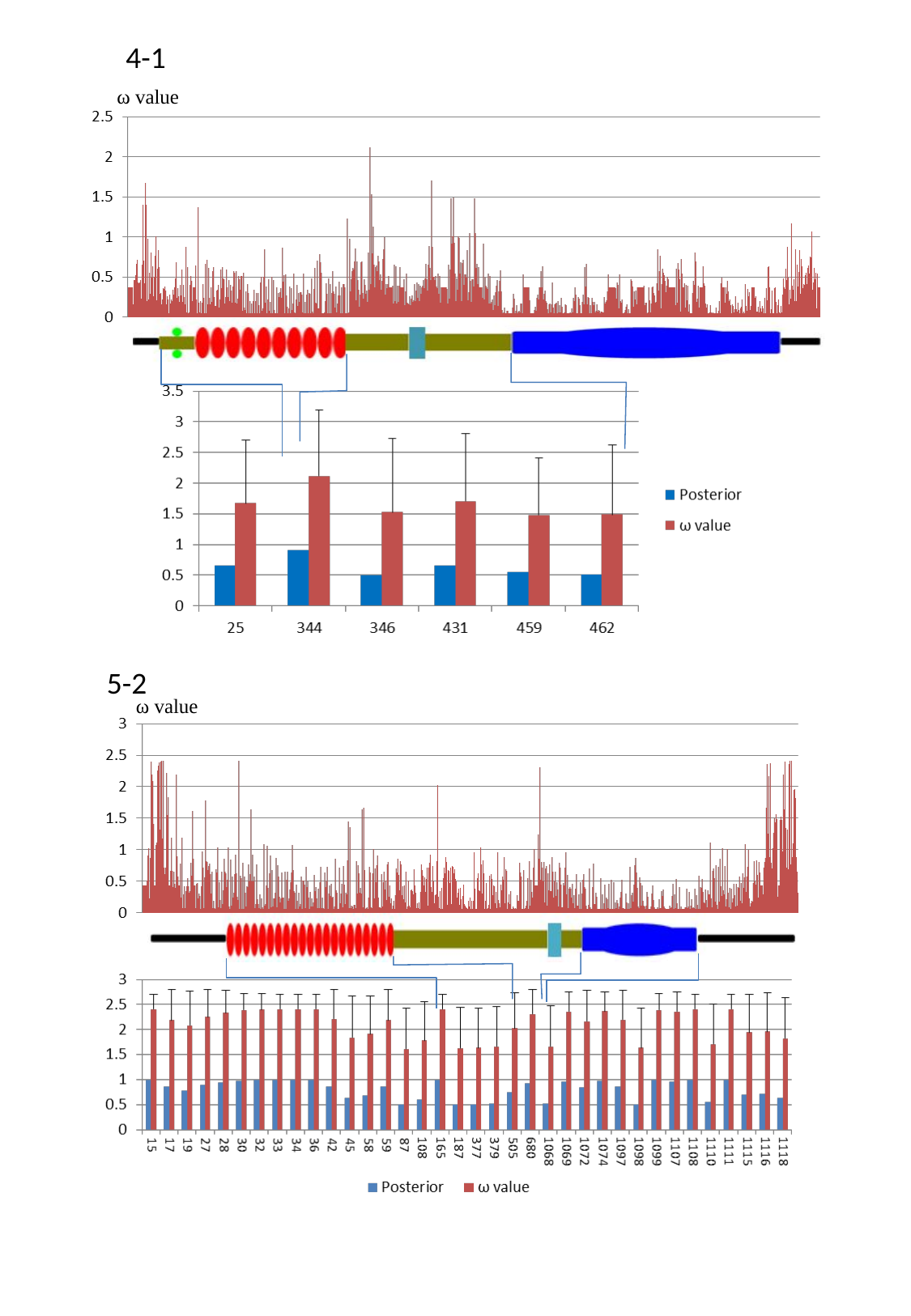

4-1
ω value
5-2
ω value

## Slide 5
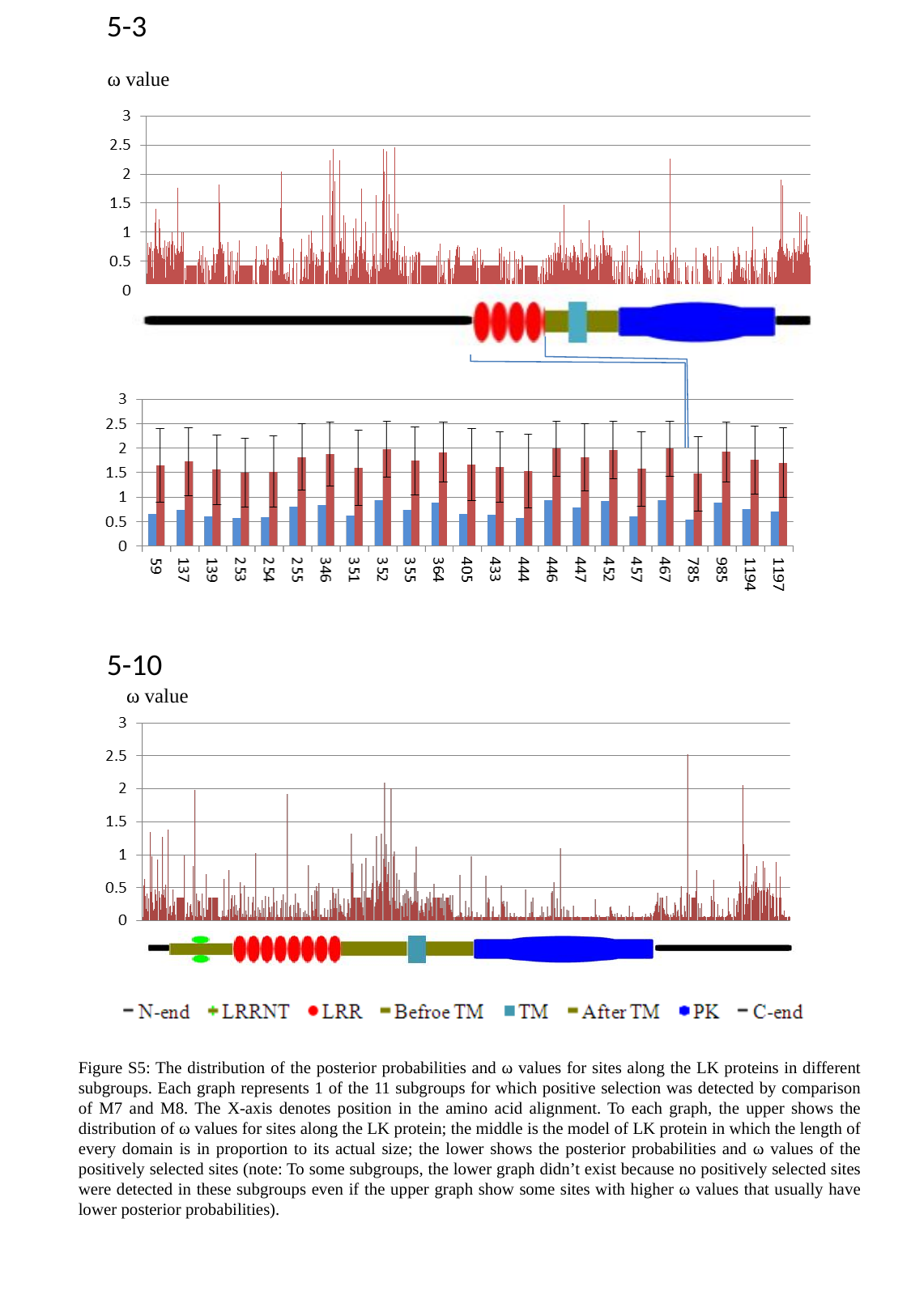

5-3
ω value
5-10
ω value
Figure S5: The distribution of the posterior probabilities and ω values for sites along the LK proteins in different subgroups. Each graph represents 1 of the 11 subgroups for which positive selection was detected by comparison of M7 and M8. The X-axis denotes position in the amino acid alignment. To each graph, the upper shows the distribution of ω values for sites along the LK protein; the middle is the model of LK protein in which the length of every domain is in proportion to its actual size; the lower shows the posterior probabilities and ω values of the positively selected sites (note: To some subgroups, the lower graph didn’t exist because no positively selected sites were detected in these subgroups even if the upper graph show some sites with higher ω values that usually have lower posterior probabilities).
