# Supplementary material for: Genome-Wide Identification, Characterization and Phylogenetic Analysis of the Rice LRR-Kinases
Source: PLoS One. 2011 Mar 8;6(3):e16079. doi: 10.1371/journal.pone.0016079 (PMC3050792; doi:10.1371/journal.pone.0016079)
Supplement: Figure S6 — The percentage differences of amino acid sites in deduced proteins of the LKs in 11 subgroups. (PPT) [file pone.0016079.s006.ppt]

## Slide 1
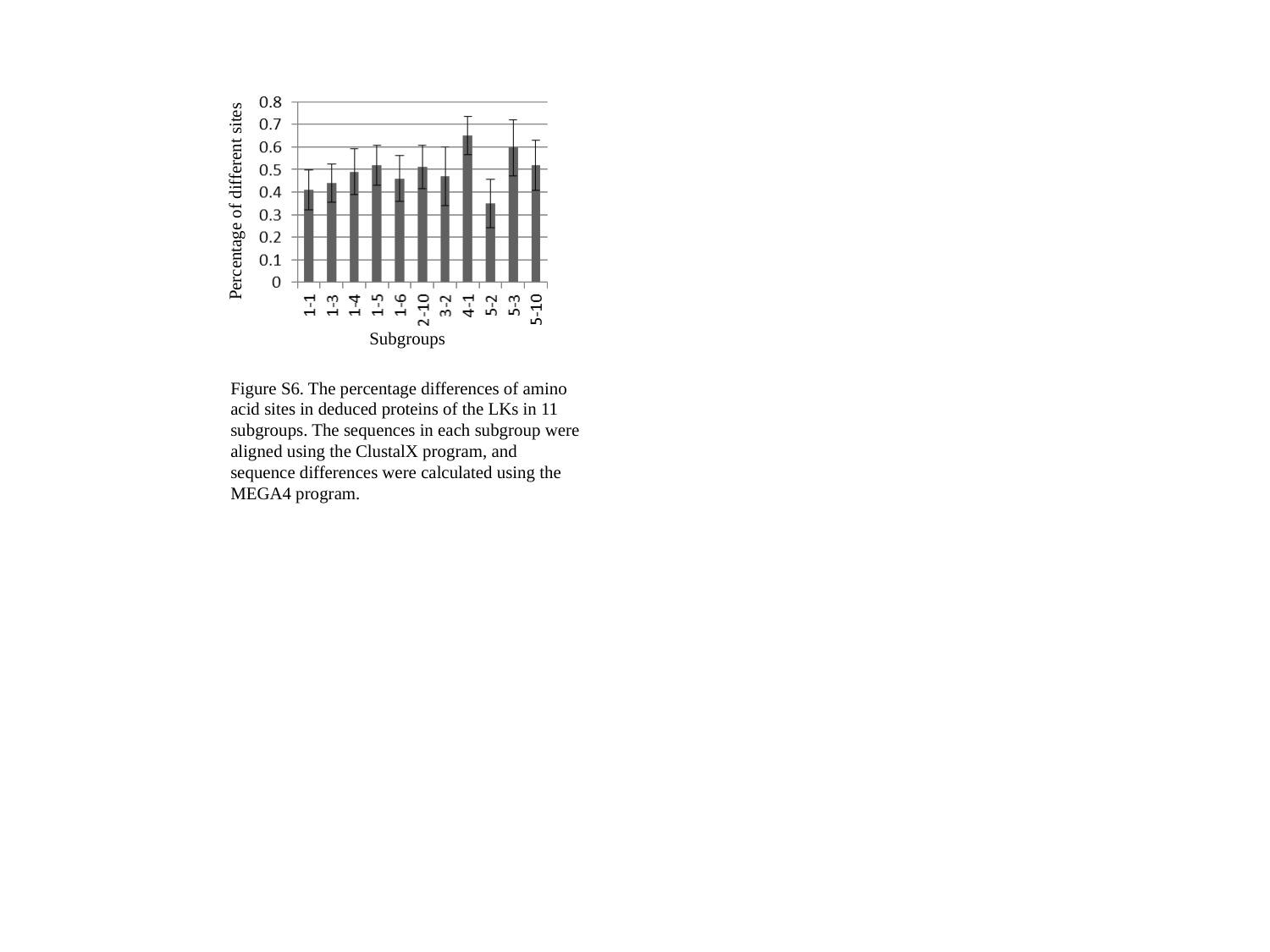

Percentage of different sites
Subgroups
Figure S6. The percentage differences of amino acid sites in deduced proteins of the LKs in 11 subgroups. The sequences in each subgroup were aligned using the ClustalX program, and sequence differences were calculated using the MEGA4 program.
