# Supplementary material for: Genome-Wide Identification, Characterization and Phylogenetic Analysis of the Rice LRR-Kinases
Source: PLoS One. 2011 Mar 8;6(3):e16079. doi: 10.1371/journal.pone.0016079 (PMC3050792; doi:10.1371/journal.pone.0016079)
Supplement: Table S3 — The functions of some known LKs and their orthologs in rice. (DOC) [file pone.0016079.s009.doc]

Table S3: The functions of some known LKs and their orthologs in rice

.

| **Name** | **Accession number** | **Source** | **Orthologs in Rice** | **Group or subgroup** | **Function** | **Reference** |
| --- | --- | --- | --- | --- | --- | --- |
| **FLS2** | AT5G46330 | *Arabidopsis* | Os04g0618700 | 1 | Flagellin perception | (Gomez-Gomez and Boller, 2000) |
| **Xa26** | DQ355952(AY364476) | rice | Os11g69470 or Os11g69230 | 1-1 | Resistance to *Xanthomonas oryzae* pv. *Oryzae* | (Sun et al., 2004) |
| **EFR** | AT5G20480 | *Arabidopsis* | Os10g033740 or Os01g0152600 | 1-4 or 1-5 | Perception of the bacterial PAMP EF-Tu | (Zipfel et al., 2006) |
| **Xa21** | U37133 | rice | Os11g0569600 or Os11g0569500 | 1-5 | Resistance to *Xanthomonas oryzae* pv. *Oryzae* | (Song et al., 1995) |
| **GSO1** | AT4G20140 | *Arabidopsis* | Os07g0498400 | 2 | Involve in normal development of the epidermal surface in Arabidopsis embryos | (Tsuwamoto et al., 2008) |
| **GSO2** | AT5G44700 | *Arabidopsis* | Os07g0498400 | 2 | Involve in normal development of the epidermal surface in Arabidopsis embryos | (Tsuwamoto et al., 2008) |
| **IKU2** | AT3G19700 | *Arabidopsis* | Os12g0632800 | 2 | Control seed size | (Garcia et al., 2003) |
| **PEPR1** | AT1G73080 | *Arabidopsis* | Os08g0446200 | 2-2 | The receptor for AtPep1 to amplify innate immunity in response to pathogen attacks | (Yamaguchi et al., 2006) |
| **ERECTA** | At2g26330 | *Arabidopsis* | Os06g0203800 | 2-3 | Determining organ shape and  resistance to bacterial wilt | (Torii et al., 1996)  (Godiard et al., 2003) |
| **BAM3** | AT4G20270 | *Arabidopsis* | Os07g0134200 | 2-6 | Development of leaf, male gametophyte and ovule | (DeYoung et al., 2006) |
| **CLV1/HAR1/NARK** | [AT1G75820](http://www.arabidopsis.org/servlets/TairObject?type=aa_sequence&id=1009105743) | *Arabidopsis* | [Os06g0717200](http://www.ncbi.nlm.nih.gov/sites/entrez?db=gene&cmd=search&term=4342080&RID=GPYHN2F7013&log$=geneexplicitprot&blast_rank=1) | 2-6 | Control of apical meristem proliferation | (Clark et al., 1997) |
| **FON1** | AB182388 | rice | [Os06g0717200](http://www.ncbi.nlm.nih.gov/sites/entrez?db=gene&cmd=search&term=4342080&RID=AXKHXCVG01S&log$=geneexplicitprot&blast_rank=1) | 2-6 | Regulation of floral organ number and floral meristem size | (Huang and Ma, 1997; Moon et al., 2006) |
| **HAESA** | AT4G28490 | *Arabidopsis* | Os01g0239700 | 2-9 | Delayed floral organ abscission | (Jinn et al., 2000) |
| **BRI1/SR160** | AT4G39400 | *Arabidopsis* | [Os01g0718300](http://www.ncbi.nlm.nih.gov/sites/entrez?db=gene&cmd=search&term=4324691&RID=GPZ0XETF013&log$=geneexplicitprot&blast_rank=1) | 3-1 | Brassinosteroid-mediated perception | (Li and Chory, 1997) |
| **BRL3** | AT3G13380 | *Arabidopsis* | Os09g0293500 | 3-1 | Brassinosteroid-mediated perception | (Cano-Delgado et al., 2004) |
| **EMS1/EXS** | AT5G07280 | *Arabidopsis* | Os01g0917500 | 3-1 | Controls somatic and reproductive cell fates in the Arabidopsis anther | (Canales et al., 2002; Zhao et al., 2002) |
| **PRK1** | At2g02220 | *Petunia inflata* | Os02g0629400 | 3-2 | Postmeiotic development of microspores | (Lee et al., 1996) |
| **PSK** | AB060167 | *D. carota* | Os02g0629400 | 3-2 | Perception of a peptide plant hormone, phytosulfokine | (Matsubayashi et al., 2002) |
| **RPK1** | AT1G69270 | *Arabidopsis* | Os07g0602700 | 4-4 | Regulator of abscisic acid early signaling | (Osakabe et al., 2005) |
| **NIK1** | AT1G60800 | *Arabidopsis* | Os06g0274500 | 5-10 | Targets of the geminivirus nuclear shuttle protein (NSP), NSP interacts with pK domain and inhibit its function | (Fontes et al., 2004) |
| **NIK2** | AT3G25560 | *Arabidopsis* | Os06g0274500 | 5-10 | Targets of the geminivirus nuclear shuttle protein (NSP), NSP interacts with pK domain and inhibit its function | (Fontes et al., 2004) |
| **NIK3** | AT1G60800 | *Arabidopsis* | Os01g0171000 | 5-10 | Targets of the geminivirus nuclear shuttle protein (NSP), NSP interacts with pK domain and inhibit its function | (Fontes et al., 2004) |
| **SERK1** | AT1G71830 | *Arabidopsis* | Os08g0174700 | 5-10 | Control Male Sporogenesis | (Hecht et al., 2001) |
| **SERK2** | AT1G34210 | *Arabidopsis* | Os08g0174700 | 5-10 | Control Male Sporogenesis | (Albrecht et al., 2005) |
| **BAK1** | AT4G33430 | *Arabidopsis* | Os08g0174700 | 5-10 | Steroid hormone–mediated growth response  initiation of innate immunity | (Li et al., 2002)  {Chinchilla, 2007 #91} |
| **SHR5** | AAY67902 | *Saccharum hybrid cultivar SP70-1143* | Os08g0203700 | 5-2 | involve in establishment of plant–endophytic bacteria interaction | (Vinagre et al., 2006) |
| **SIRK** | At2g19190 | *Arabidopsis* | Os05g0525600 | 5-3 | Upregulated during senescence and pathogen challenge | (Robatzek and Somssich, 2002) |
| **NORK/SYMRK** | Swiss-Prot:Q8L4H4.2 | *Medicago trunculata* | Os07g0568100 | 5-3 | Development of root nodule | (Endre et al., 2002; Stracke et al., 2002) |
| **SCM** | AT1G11130 | *Arabidopsis* | Os02g0136900 | 5-8 | Positional signaling in developing root epidermis | (Kwak et al., 2005) |

**Reference**

**Albrecht C, Russinova E, Hecht V, Baaijens E, de Vries S** (2005) The Arabidopsis thaliana SOMATIC EMBRYOGENESIS RECEPTOR-LIKE KINASES1 and 2 control male sporogenesis. Plant Cell **17:** 3337-3349

**Canales C, Bhatt AM, Scott R, Dickinson H** (2002) EXS, a putative LRR receptor kinase, regulates male germline cell number and tapetal identity and promotes seed development in Arabidopsis. Curr Biol **12:** 1718-1727

**Cano-Delgado A, Yin Y, Yu C, Vafeados D, Mora-Garcia S, Cheng JC, Nam KH, Li J, Chory J** (2004) BRL1 and BRL3 are novel brassinosteroid receptors that function in vascular differentiation in Arabidopsis. Development **131:** 5341-5351

**Clark SE, Williams RW, Meyerowitz EM** (1997) The CLAVATA1 gene encodes a putative receptor kinase that controls shoot and floral meristem size in Arabidopsis. Cell **89:** 575-585

**DeYoung BJ, Bickle KL, Schrage KJ, Muskett P, Patel K, Clark SE** (2006) The CLAVATA1-related BAM1, BAM2 and BAM3 receptor kinase-like proteins are required for meristem function in Arabidopsis. Plant J **45:** 1-16

**Endre G, Kereszt A, Kevei Z, Mihacea S, Kalo P, Kiss GB** (2002) A receptor kinase gene regulating symbiotic nodule development. Nature **417:** 962-966

**Fontes EP, Santos AA, Luz DF, Waclawovsky AJ, Chory J** (2004) The geminivirus nuclear shuttle protein is a virulence factor that suppresses transmembrane receptor kinase activity. Genes Dev **18:** 2545-2556

**Garcia D, Saingery V, Chambrier P, Mayer U, Jurgens G, Berger F** (2003) Arabidopsis haiku mutants reveal new controls of seed size by endosperm. Plant Physiol **131:** 1661-1670

**Godiard L, Sauviac L, Torii KU, Grenon O, Mangin B, Grimsley NH, Marco Y** (2003) ERECTA, an LRR receptor-like kinase protein controlling development pleiotropically affects resistance to bacterial wilt. Plant J **36:** 353-365

**Gomez-Gomez L, Boller T** (2000) FLS2: an LRR receptor-like kinase involved in the perception of the bacterial elicitor flagellin in Arabidopsis. Mol Cell **5:** 1003-1011

**Hanks SK, Hunter T** (1995) Protein kinases 6. The eukaryotic protein kinase superfamily: kinase (catalytic) domain structure and classification. Faseb J **9:** 576-596

**Hecht V, Vielle-Calzada JP, Hartog MV, Schmidt ED, Boutilier K, Grossniklaus U, de Vries SC** (2001) The Arabidopsis SOMATIC EMBRYOGENESIS RECEPTOR KINASE 1 gene is expressed in developing ovules and embryos and enhances embryogenic competence in culture. Plant Physiol **127:** 803-816

**Huang H, Ma H** (1997) FON1, an Arabidopsis gene that terminates floral meristem activity and controls flower organ number. Plant Cell **9:** 115-134

**Jinn TL, Stone JM, Walker JC** (2000) HAESA, an Arabidopsis leucine-rich repeat receptor kinase, controls floral organ abscission. Genes Dev **14:** 108-117

**Kwak SH, Shen R, Schiefelbein J** (2005) Positional signaling mediated by a receptor-like kinase in Arabidopsis. Science **307:** 1111-1113

**Lee HS, Karunanandaa B, McCubbin A, Gilroy S, Kao T-H** (1996) PRK1, a receptor-like kinase of Petunia inflata, is essential for postmeiotic development of pollen. The Plant J **9:** 613-624

**Li J, Chory J** (1997) A putative leucine-rich repeat receptor kinase involved in brassinosteroid signal transduction. Cell **90:** 929-938

**Li J, Wen J, Lease KA, Doke JT, Tax FE, Walker JC** (2002) BAK1, an Arabidopsis LRR receptor-like protein kinase, interacts with BRI1 and modulates brassinosteroid signaling. Cell **110:** 213-222

**Matsubayashi Y, Ogawa M, Morita A, Sakagami Y** (2002) An LRR receptor kinase involved in perception of a peptide plant hormone, phytosulfokine. Science **296:** 1470-1472

**Moon S, Jung KH, Lee DE, Lee DY, Lee J, An K, Kang HG, An G** (2006) The rice FON1 gene controls vegetative and reproductive development by regulating shoot apical meristem size. Mol Cells **21:** 147-152

**Osakabe Y, Maruyama K, Seki M, Satou M, Shinozaki K, Yamaguchi-Shinozaki K** (2005) Leucine-rich repeat receptor-like kinase1 is a key membrane-bound regulator of abscisic acid early signaling in Arabidopsis. Plant Cell **17:** 1105-1119

**Robatzek S, Somssich IE** (2002) Targets of AtWRKY6 regulation during plant senescence and pathogen defense. Genes Dev **16:** 1139-1149

**Song WY, Wang GL, Chen LL, Kim HS, Pi LY, Holsten T, Gardner J, Wang B, Zhai WX, Zhu LH, Fauquet C, Ronald P** (1995) A receptor kinase-like protein encoded by the rice disease resistance gene, Xa21. Science **270:** 1804-1806

**Stracke S, Kistner C, Yoshida S, Mulder L, Sato S, Kaneko T, Tabata S, Sandal N, Stougaard J, Szczyglowski K, Parniske M** (2002) A plant receptor-like kinase required for both bacterial and fungal symbiosis. Nature **417:** 959-962

**Sun X, Cao Y, Yang Z, Xu C, Li X, Wang S, Zhang Q** (2004) Xa26, a gene conferring resistance to Xanthomonas oryzae pv. oryzae in rice, encodes an LRR receptor kinase-like protein. Plant J **37:** 517-527

**Torii KU, Mitsukawa N, Oosumi T, Matsuura Y, Yokoyama R, Whittier RF, Komeda Y** (1996) The Arabidopsis ERECTA gene encodes a putative receptor protein kinase with extracellular leucine-rich repeats. Plant Cell **8:** 735-746

**Tsuwamoto R, Fukuoka H, Takahata Y** (2008) GASSHO1 and GASSHO2 encoding a putative leucine-rich repeat transmembrane-type receptor kinase are essential for the normal development of the epidermal surface in Arabidopsis embryos. Plant J **54:** 30-42

**Vinagre F, Vargas C, Schwarcz K, Cavalcante J, Nogueira EM, Baldani JI, Ferreira PC, Hemerly AS** (2006) SHR5: a novel plant receptor kinase involved in plant-N2-fixing endophytic bacteria association. J Exp Bot **57:** 559-569

**Yamaguchi Y, Pearce G, Ryan CA** (2006) The cell surface leucine-rich repeat receptor for AtPep1, an endogenous peptide elicitor in Arabidopsis, is functional in transgenic tobacco cells. Proc Natl Acad Sci U S A **103:** 10104-10109

**Zhao DZ, Wang GF, Speal B, Ma H** (2002) The excess microsporocytes1 gene encodes a putative leucine-rich repeat receptor protein kinase that controls somatic and reproductive cell fates in the Arabidopsis anther. Genes Dev **16:** 2021-2031

**Zipfel C, Kunze G, Chinchilla D, Caniard A, Jones JD, Boller T, Felix G** (2006) Perception of the bacterial PAMP EF-Tu by the receptor EFR restricts Agrobacterium-mediated transformation. Cell **125:** 749-760
