# Supplementary material for: Genome-Wide Identification, Characterization and Phylogenetic Analysis of the Rice LRR-Kinases
Source: PLoS One. 2011 Mar 8;6(3):e16079. doi: 10.1371/journal.pone.0016079 (PMC3050792; doi:10.1371/journal.pone.0016079)
Supplement: Table S4 — Clusters of LK genes in rice Nipponbare. (DOC) [file pone.0016079.s010.doc]

Table S4: Clusters of LK genes in rice Nipponbare

| **Group** | **No. of Clusters** | **No. of genes in clusters** | **total** | **Percent of genes in clusters** |
| --- | --- | --- | --- | --- |
| **1** | 14 | 75 | 99 | 75.7% |
| **2** | 9 | 19 | 51 | 37.3% |
| **3** | 2 | 13 | 32 | 40.6% |
| **4** |  |  | 41 |  |
| **5** | 7 | 32 | 86 | 37.2% |
| **Total** | 32 | 139 | 309 | 45.0% |
| **Chromosome** |  |  |  |  |
| **1** | 3 | 9 | 35 | 25.7% |
| **2** | 6 | 26 | 44 | 59.1% |
| **3** |  |  | 20 |  |
| **4** | 2 | 8 | 21 | 38.1% |
| **5** | 2 | 12 | 22 | 54.5% |
| **6** | 4 | 18 | 35 | 51.4% |
| **7** |  |  | 19 |  |
| **8** | 4 | 12 | 25 | 48.0% |
| **9** | 1 | 5 | 14 | 35.7% |
| **10** | 5 | 10 | 16 | 62.5% |
| **11** | 4 | 37 | 49 | 75.5% |
| **12** | 1 | 2 | 9 | 22.2% |
| **Total** | 32 | 139 | 309 | 45.0% |
